# Perturbations in Gut Microbiota Composition in Psychiatric Disorders: A Review and Meta-analysis

**DOI:** 10.1001/jamapsychiatry.2021.2573

**Published:** 2021-09-15

**Authors:** Viktoriya L. Nikolova, Megan R. B. Hall, Lindsay J. Hall, Anthony J. Cleare, James M. Stone, Allan H. Young

**Affiliations:** 1 Centre for Affective Disorders, Institute of Psychiatry, Psychology & Neuroscience, King’s College London, London, United Kingdom; 2 Department of Psychosis Studies, Institute of Psychiatry, Psychology and Neuroscience, King’s College of London, London, United Kingdom; 3 Quadram Institute Bioscience, Norwich Research Park, Norwich, United Kingdom; 4 Norwich Medical School, University of East Anglia, Norwich Research Park, Norwich, United Kingdom; 5 Chair of Intestinal Microbiome, School of Life Sciences, ZIEL–Institute for Food & Health, Technical University of Munich, Freising, Germany; 6 National Institute for Health Research Biomedical Research Centre at South London and Maudsley NHS Foundation Trust, King’s College London, London, United Kingdom; 7 South London and Maudsley NHS Foundation Trust, Bethlem Royal Hospital, Beckenham, United Kingdom; 8 Brighton and Sussex Medical School, Brighton, United Kingdom

## Abstract

**Importance:**

Evidence of gut microbiota perturbations has accumulated for multiple psychiatric disorders, with microbiota signatures proposed as potential biomarkers. However, no attempts have been made to evaluate the specificity of these across the range of psychiatric conditions.

**Objective:**

To conduct an umbrella and updated meta-analysis of gut microbiota alterations in general adult psychiatric populations and perform a within- and between-diagnostic comparison.

**Data Sources:**

Cochrane Library, PubMed, PsycINFO, and Embase were searched up to February 2, 2021, for systematic reviews, meta-analyses, and original evidence.

**Study Selection:**

A total of 59 case-control studies evaluating diversity or abundance of gut microbes in adult populations with major depressive disorder, bipolar disorder, psychosis and schizophrenia, anorexia nervosa, anxiety, obsessive compulsive disorder, posttraumatic stress disorder, or attention-deficit/hyperactivity disorder were included.

**Data Extraction and Synthesis:**

Between-group comparisons of relative abundance of gut microbes and beta diversity indices were extracted and summarized qualitatively. Random-effects meta-analyses on standardized mean difference (SMD) were performed for alpha diversity indices.

**Main Outcomes and Measures:**

Alpha and beta diversity and relative abundance of gut microbes.

**Results:**

A total of 34 studies provided data and were included in alpha diversity meta-analyses (n = 1519 patients, n = 1429 control participants). Significant decrease in microbial richness in patients compared with control participants were found (observed species SMD = −0.26; 95% CI, −0.47 to −0.06; Chao1 SMD = −0.5; 95% CI, −0.79 to −0.21); however, this was consistently decreased only in bipolar disorder when individual diagnoses were examined. There was a small decrease in phylogenetic diversity (SMD = −0.24; 95% CI, −0.47 to −0.001) and no significant differences in Shannon and Simpson indices. Differences in beta diversity were consistently observed only for major depressive disorder and psychosis and schizophrenia. Regarding relative abundance, little evidence of disorder specificity was found. Instead, a transdiagnostic pattern of microbiota signatures was found. Depleted levels of *Faecalibacterium* and *Coprococcus* and enriched levels of *Eggerthella* were consistently shared between major depressive disorder, bipolar disorder, psychosis and schizophrenia, and anxiety, suggesting these disorders are characterized by a reduction of anti-inflammatory butyrate-producing bacteria, while pro-inflammatory genera are enriched. The confounding associations of region and medication were also evaluated.

**Conclusions and Relevance:**

This systematic review and meta-analysis found that gut microbiota perturbations were associated with a transdiagnostic pattern with a depletion of certain anti-inflammatory butyrate-producing bacteria and an enrichment of pro-inflammatory bacteria in patients with depression, bipolar disorder, schizophrenia, and anxiety.

## Key Points

**Question:**

Do psychiatric disorders present with distinct or shared gut microbial alterations?

**Findings:**

This review and meta-analysis of 59 case-control studies found that gut microbiota perturbations were associated with a transdiagnostic pattern with a depletion of certain anti-inflammatory butyrate-producing bacteria and an enrichment of pro-inflammatory bacteria in depression, bipolar disorder, schizophrenia, and anxiety.

**Meaning:**

These findings are in line with genetic and inflammatory marker studies and support the transdiagnostic dimensional model of psychiatric disorders by highlighting the gut microbiota as an additional dimensional component.

## Teaser

This systematic review and meta-analysis reviews gut microbiota alterations in general adult psychiatric populations and performs a within- and between-diagnostic comparison.

## Introduction

Despite evidence that probiotic formulations can improve mental health dating back to the early 20th century,^1,2^ it was only following advances in DNA/RNA sequencing technologies that the involvement of the gut microbiota in the pathophysiology of psychiatric disorders was recognized. Preclinical studies have consistently demonstrated that fecal microbiota transplants from patients with a wide range of psychiatric conditions result in the development of the behavioral and physiological profile of the condition in germ-free mice.^3,4,5,6,7^ This suggests that psychiatric disorders may be associated with a distinct pattern of microbial perturbations, which may serve as a biomarker.

Attempts to characterize the composition of the microbiota in psychiatric populations have yielded plentiful yet contradictory results. Nevertheless, systematic reviews in individual disorders have been able to identify patterns that may be promising biomarker targets.^8,9,10^ Indeed, the addition of such biomarkers can improve diagnostic accuracy, guide treatment, and assist the monitoring of treatment response. For the definition of a biomarker to be met, ie, “substance, structure or process that can be measured in the body and influence or predict the incidence of outcome or disease,”^11^ the specificity and reproducibility of the alteration needs to be demonstrated.^12^ Therefore, it is crucial to compare microbial perturbations across the wider range of psychiatric conditions.

We performed an umbrella and updated review and meta-analysis of gut microbiota studies in adults with major depressive disorder (MDD), bipolar disorder, psychosis and schizophrenia, anxiety disorders, obsessive compulsive disorder (OCD), eating disorders (anorexia nervosa and bulimia nervosa), autism spectrum disorder, attention-deficit/hyperactivity disorder (ADHD), and posttraumatic stress disorder (PTSD) to evaluate the specificity and reproducibility of gut microbiota alterations and delineate those with potential to become biomarkers.

## Methods

The protocol for this review was preregistered with PROSPERO (CRD42021224342). We followed Preferred Reporting Items for Systematic Reviews and Meta-analyses (PRISMA) reporting guideline^13^ as well as Cochrane guidance for umbrella and updated reviews.^14,15^

### Search Details

We searched Cochrane Library, PubMed, Embase, and PsycINFO on January 27, 2021. The search strings used are available in eAppendix 1 in the Supplement. This search was limited to systematic reviews and meta-analyses in English, including human studies, published since 2005. After reviewing the results, we realized that a large body of recent literature was missed, as numerous studies have become available following the publication of the latest reviews. To ensure thorough coverage, we performed an updated search for each disorder on February 2, 2021, from the search date recorded in the latest available high-quality review for that disorder (eAppendix 1 in the Supplement).

### Selection Criteria

Systematic reviews and meta-analyses were considered eligible if they followed established guidelines and included at least 1 eligible original study. Original studies were eligible if they (1) applied an observational case-control design, (2) performed gut microbiota analysis and reported diversity or abundance measures, and (3) sampled a general adult population (age 18-65 years) with a psychiatric diagnosis of interest. Interventional or longitudinal comparisons in the absence of a control group were excluded. Records were screened by 2 authors (V.L.N. and M.R.B.S) and discrepancies resolved via discussion and consultation with a third author (A.H.Y.).

### Data Extraction

Information was extracted using a predesigned template by 2 authors (V.L.N. and M.R.B.S) and cross-checked. From systematic reviews and original studies, we extracted publication details, participant demographic and clinical characteristics, and methodological information. As primary outcomes of interest, we extracted community-level measures of gut microbiota composition (alpha and beta diversity) and taxonomic findings at the phylum, family, and genus levels (relative abundance). Alpha diversity provides a summary of the microbial community in individual samples and can be compared across groups to evaluate the role of a particular factor (in this case psychiatric diagnosis) on the richness (number of species) and evenness (how well each species is represented) in the sample.^10,16^ Beta diversity is a measure of interindividual (between samples) diversity that assesses similarity of communities compared with the other samples analyzed.^10^ This analysis allows us to see whether patient samples cluster significantly differently (ie, with little or no overlap) compared with control participant samples or whether they overlap, thus suggesting the 2 groups are not distinct. Control samples were defined as individuals without the relevant condition.

### Quality Assessment

We performed quality assessment of the systematic reviews using the ROBIS tool^17^ and of the original studies not covered in any review with the Joanna Briggs Institute Critical Appraisal Checklist for Case-Control Studies.^18^ No studies were excluded owing to quality concerns. The detailed assessment is available as eAppendix 2 in the Supplement.

### Qualitative Synthesis

For the relative abundance of microbial taxa, we performed a qualitative synthesis owing to the large number and limited overlap of findings. Owing to the significant likelihood of false positives noted in previous meta-analyses,^19^ results reported only by a single study were excluded. Further, results reported only by 1 research group were also excluded because these were considered potentially methodology or population specific. To identify disease-specific and shared alterations, we performed a within- and between-diagnostic comparison. First, we summarized within-disorder findings for each taxon reported in at least 2 studies and labeled those increased, decreased, or not consistent. Not consistent was any finding with less than 75% agreement between studies reporting this taxon. A consistent finding by 2 studies was considered worth noting for future validation, whereas a finding by 3 or more studies (from ≥2 research groups) was considered potentially associated with the disorder. A taxon was considered a candidate for disease-specific response if it was altered (in a consistent direction) in a single disorder only. Alternatively, if a shift was replicated in several disorders with known symptomatic and pathophysiological overlap, this was considered a transdiagnostic alteration. Taxa similarly altered across all/multiple unrelated diagnostic categories were interpreted as general disease response.

### Quantitative Synthesis

Meta-analysis was performed on differences in alpha diversity between patients and controls for indices with data reported in 10 or more studies. Detailed methods of data transformation and interpretation thresholds are available in eAppendix 3 in the Supplement. Publication bias was evaluated with funnel plots and Egger test. Preplanned subgroup analyses were disorder, region of study (east/west), and use of psychiatric medication. All analyses were completed in R version 4.17-0 (*meta* package; R Foundation).^20^ Two-sided *P *values were statistically significant at less than .05.

## Results

### Search Results

We identified 16 systematic reviews (eAppendices 4 and 5 in the Supplement for PRISMA flowcharts and details of the systematic reviews) containing 39 eligible studies. There were no reviews capturing OCD, PTSD, or autism spectrum disorder in adults. In the second search, a further 20 studies were identified, resulting in 59 studies across 8 disorders. The most researched disorder was MDD, followed by psychosis and schizophrenia, bipolar disorder, and anorexia nervosa (Table).

**Table.** Summary Characteristics of the Identified Reviews and Original Studies by Psychiatric Disorder

Disorder^a^	No.^b^			Region of studies^c^	Mean patient age, y	Female, mean %
	Reviews	Studies	Total patients			
MDD	8	21	930	East: n = 14; west: n = 7	35	60
Schizophrenia and psychosis	5	11	699	East: n = 9; west: n = 2	36	45
Bipolar disorder	3	9	465	East: n = 5; west: n = 4	38	55
Anorexia nervosa	3	10	211	East: n = 2; west: n = 8	26	99
Anxiety	2	3	84	East: n = 2; west: n = 1	40	77
OCD	0	2	59	West: n = 2	36	54
PTSD	0	1	18	Africa: n = 1	42	14
ADHD^d^	1	1	19	West: n = 1	20	32
MDD + anxiety	NA	2	60	West: n = 2	39	82
MDD + bipolar disorder	NA	2	98	East: n = 1; west: n = 1	37	69
Total	16	59	2643	East: n = 32; west: n = 24; Africa: n = 1	NA	NA

Abbreviations: ADHD, attention-deficit/hyperactivity disorder; MDD, major depressive disorder; NA, not applicable; OCD, obsessive compulsive disorder; PTSD, posttraumatic stress disorder.

^a^ Studies that examined combined cohorts (MDD + bipolar disorder^21,22^ or MDD + anxiety^23,24^) are presented separately.

^b^ Some include >1 disorder.

^c^ West region includes US, Canada, Europe, Australia, and New Zealand. East region includes China, Japan, and Taiwan. Africa includes South Africa.

^d^ Adult populations only.

### Characteristics of Included Studies

The 59 studies provided 64 case-control comparisons capturing 2643 patients and 2336 controls (eAppendix 6 in the Supplement provides a detailed summary of study characteristics). Most studies (32 [54.2%]) were conducted in East Asia (China, Japan, and Taiwan), 24 (40.7%) in westernized populations (US, Canada, Europe, Australia, and New Zealand; grouped according to typical diet and lifestyle), and 1 (1.7%) in Africa (South Africa). Most studies had small to moderate sample sizes (median, 62), ranging between 4 and 156 per group (eAppendix 6 in the Supplement). Studies were similar in exclusion criteria; however, few attempted to minimize dietary changes or control dietary intake (12 of 59 [20.3%]) or smoking status (8 of 59 [13.6%]). Use of psychiatric medication also varied substantially, with 11 of 59 studies (18.6%) conducted in medication-free or drug-naive groups, 5 of 59 (8.5%) in groups undergoing treatment and the remainder not controlling this, resulting in anywhere between 20% and 96% of patients taking medication. Methodology of stool processing (eAppendix 7 in the Supplement) and composition analysis (eAppendix 6 in the Supplement) also varied widely, with 16S ribosomal RNA sequencing being most common (44 of 59 studies [74.6%]) followed by 9 studies (15.2%) using quantitative polymerase chain reaction or real-time quantitative polymerase chain reaction and 7 (11.9%) using shotgun metagenomics.

### Alpha Diversity

Of 44 studies reporting alpha diversity, 34 provided data and were included in meta-analyses (1519 patients and 1429 controls). Eleven indices were used to assess alpha diversity, including estimates of richness (observed species, Chao1, abundance coverage estimator, and incidence coverage estimator), evenness, richness/evenness (Shannon, Simpson, inverse Simpson, Fisher), biodiversity (Faith phylogenetic diversity), and 1 newly developed index^25^ (eAppendix 6 in the Supplement). The most widely used were observed species, Chao1, Shannon, Simpson, and phylogenetic diversity. There was no evidence of publication bias in any of the analyses (eAppendix 8 in the Supplement).

**Figure 1.**
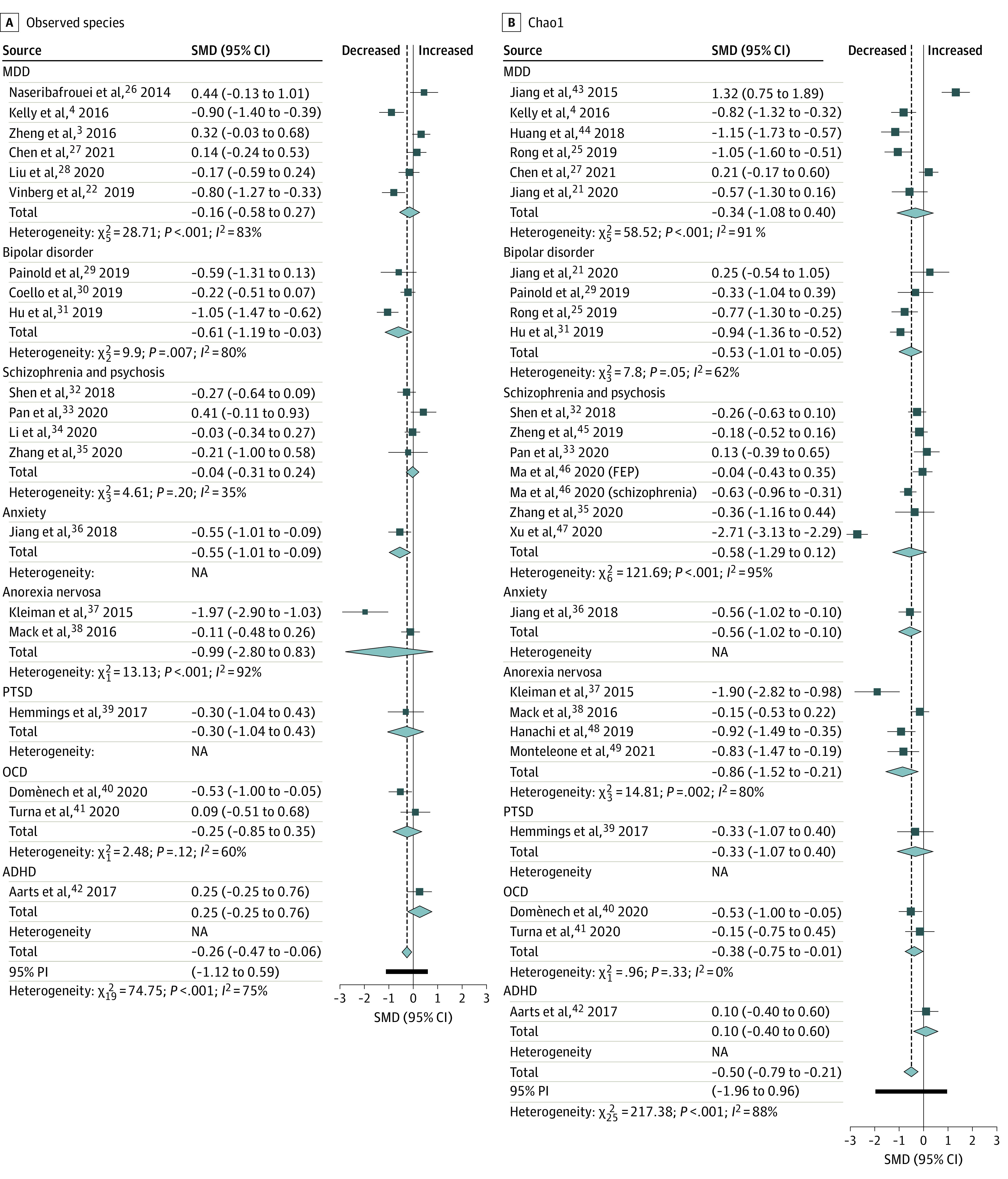
Forest Plots of Alpha Diversity Richness Estimators in the Gut Microbiota of Patients With Psychiatric Disorders Compared With Healthy Controls ADHD indicates attention-deficit/hyperactivity disorder; FEP, first episode psychosis; MDD, major depressive disorder; NA, not applicable; OCD, obsessive compulsive disorder; PI, prediction interval; PTSD, posttraumatic stress disorder; SMD, standardized mean difference.

Regarding richness, 20 studies provided data on observed species in patients (n = 897) vs controls (n = 789). The pooled estimate showed a significant decrease in patients with a small effect size (standardized mean difference [SMD] = −0.26; 95% CI, −0.47 to −0.06; *P* = .01) and high heterogeneity (*I*^2^ = 75%) (Figure 1A).^3,4,22,26,27,28,29,30,31,32,33,34,35,36,37,38,39,40,41,42^ Within diagnostic categories, there was a significant decrease only in bipolar disorder (SMD = −0.61; 95% CI, −1.19 to −0.03; *P* = .04; *I*^2^ = 80%). Twenty-six studies provided data on Chao1 in patients (n = 956) vs controls (n = 961). The pooled estimate showed a significant decrease in patients with a medium effect size (SMD = −0.5; 95% CI, −0.79 to −0.21; *P* = .001; *I*^2^ = 88%). Regarding individual diagnoses, there was a significant decrease only in bipolar disorder and anorexia nervosa (SMD = −0.53; 95% CI, −1.01 to −0.05; *P* = .03; *I*^2^ = 62% and SMD = −0.86; 95% CI, −1.52 to −0.21; *P* = .01; *I*^2^ = 80%, respectively) (Figure 1B).^4,21,25,27,29,31,32,33,35,36,37,38,39,40,41,42,43,44,45,46,47,48,49^

**Figure 2.**
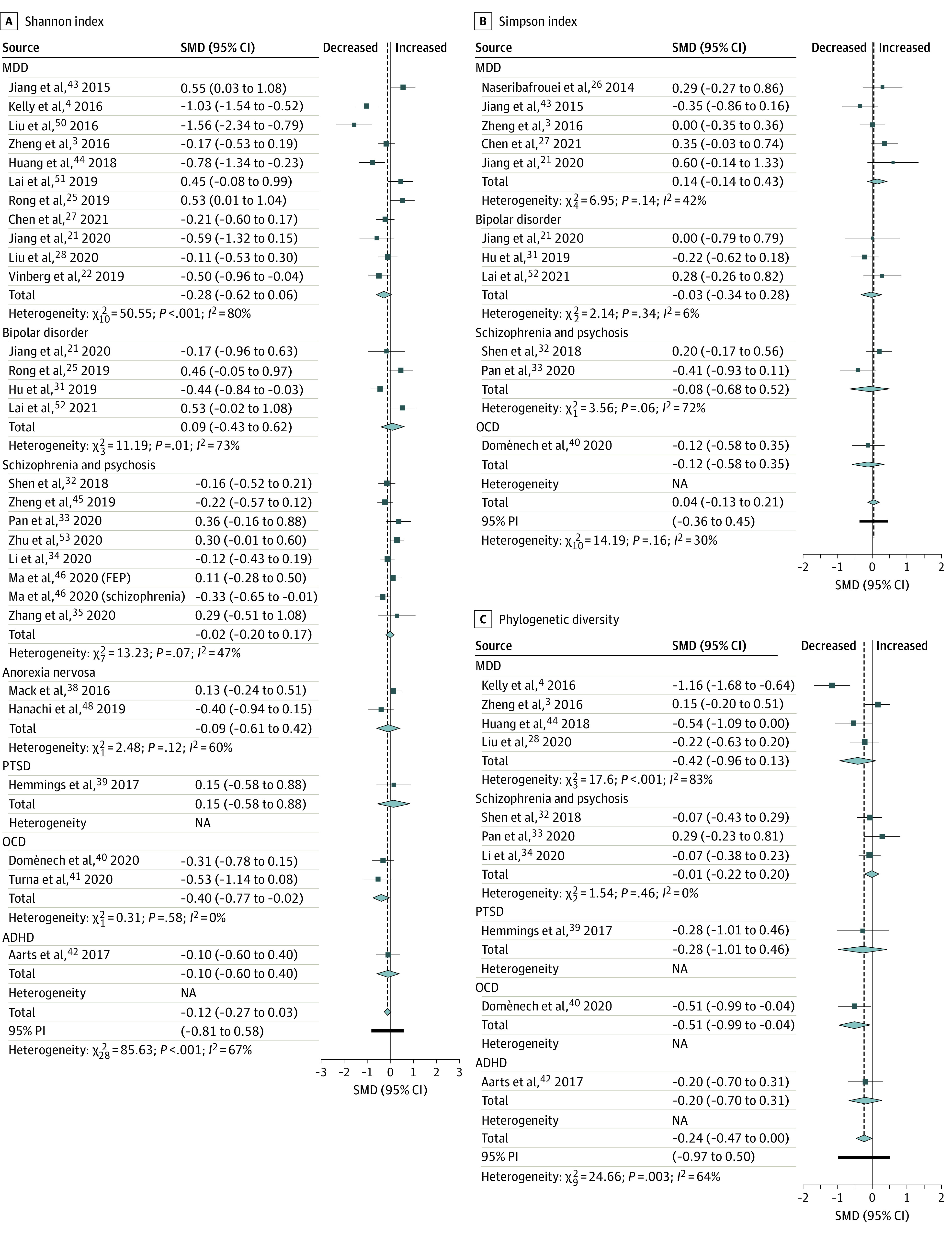
Forest Plots of Alpha Diversity in the Gut Microbiota of Patients With Psychiatric Disorders Compared With Healthy Controls ADHD indicates attention-deficit/hyperactivity disorder; FEP, first episode psychosis; MDD, major depressive disorder; NA, not applicable; OCD, obsessive compulsive disorder; PI, prediction interval; PTSD, posttraumatic stress disorder; SMD, standardized mean difference.

Regarding diversity, 29 studies reported the Shannon index in patients (n = 1176) vs controls (n = 1172). The pooled estimate demonstrated a nonsignificant difference between groups (SMD = −0.12; 95% CI, −0.27 to 0.03; *P* = .11) (Figure 2A).^3,4,21,22,25,27,28,31,32,33,34,35,38,39,40,41,42,43,44,45,46,48,50,51,52,53^ Simpson index data were provided by 11 studies (n = 418 patients; n = 377 controls). There was a nonsignificant difference between groups (SMD = 0.04; 95% CI, −0.13 to 0.21; *P* = .66), with nonsignificant heterogeneity (Figure 2B).^21,26,27,31,32,33,40,43,52^ Finally, 10 studies provided phylogenetic diversity data in patients (n = 412) vs controls (n = 454). The pooled estimate showed a significant decrease in patients with a small effect size (SMD = −0.24; 95% CI, −0.47 to −0.0012; *P* = .049; 64%) (Figure 2C).^3,4,28,32,33,34,39,40,42,44^

To explore sources of interstudy heterogeneity, subgroup analyses and meta-regressions were performed for the analyses with sufficient studies (observed species, Chao1, Shannon). Body mass index, age, sex, smoking, region (east/west), psychiatric medication use, subgrouping of psychosis and schizophrenia into first episode, and chronic and sequencing method (including hypervariable region sequenced) did not have a significant association with findings. However, it should be noted that shotgun metagenomics showed increased Shannon diversity in patients (4 studies) in comparison with 16SrRNA V3-V4 sequencing, which showed an overall decrease (12 studies). This could be because the shotgun approach quantifies all genomic DNA (including mycobiome and virome) rather than just specific regions of bacterial DNA. Further studies using shotgun metagenomics or comparing the 2 methodologies on the same population are needed.

### Beta Diversity

Beta diversity comparison between patients and controls was reported in 43 studies, with 1 study reporting on 3 separate groups (MDD, anxiety, and MDD + anxiety^23^), using a variety of measures (eAppendix 9 in the Supplement). Consistent nonsignificant differences were reported by 16 studies, and a further 3 reported conflicting results between the measures used. Patients’ samples clustered differently from controls in 12 of 15 studies in MDD, 7 of 9 in psychosis and schizophrenia, 3 of 6 in bipolar disorder, 3 of 6 in anorexia nervosa, 2 of 3 in anxiety, 0 of 2 in OCD, and 0 of 1 in PTSD (eAppendix 9 in the Supplement). One of 2 combined MDD + bipolar disorder cohort was also significantly different from controls, whereas the MDD + anxiety cohort was not. Although, while Mason et al^23^ found no differences when looking at diagnostic categories, they found a significant difference when clustering participants according to self-reported symptoms. These findings suggest there is reliable evidence for differences in the shared phylogenetic structure in MDD and psychosis and schizophrenia compared with controls; however, method of measurement and method of patient classification (symptom vs diagnosis based) may affect findings.

### Differentially Abundant Microbial Taxa

All studies assessed the relative abundance of gut microbes and 57 of 59 (96.6%) identified significant differences between patients and controls at phylum, family, or genus levels. Overall, in MDD (21 comparisons), 94 taxa were differentially abundant; in psychosis and schizophrenia (11 comparisons), 136; in bipolar disorder (9 comparisons), 60; in anxiety (2 comparisons), 36; in anorexia nervosa (10 comparisons), 32; in OCD (2 comparisons), 15; and in ADHD and PTSD (1 study each), 9 and 3, respectively. After removal of nonreplicated findings, the differences spanned 7 phyla, 28 families, and 67 genera. Study-level findings are presented in eAppendix 10 in the Supplement.

**Figure 3.**
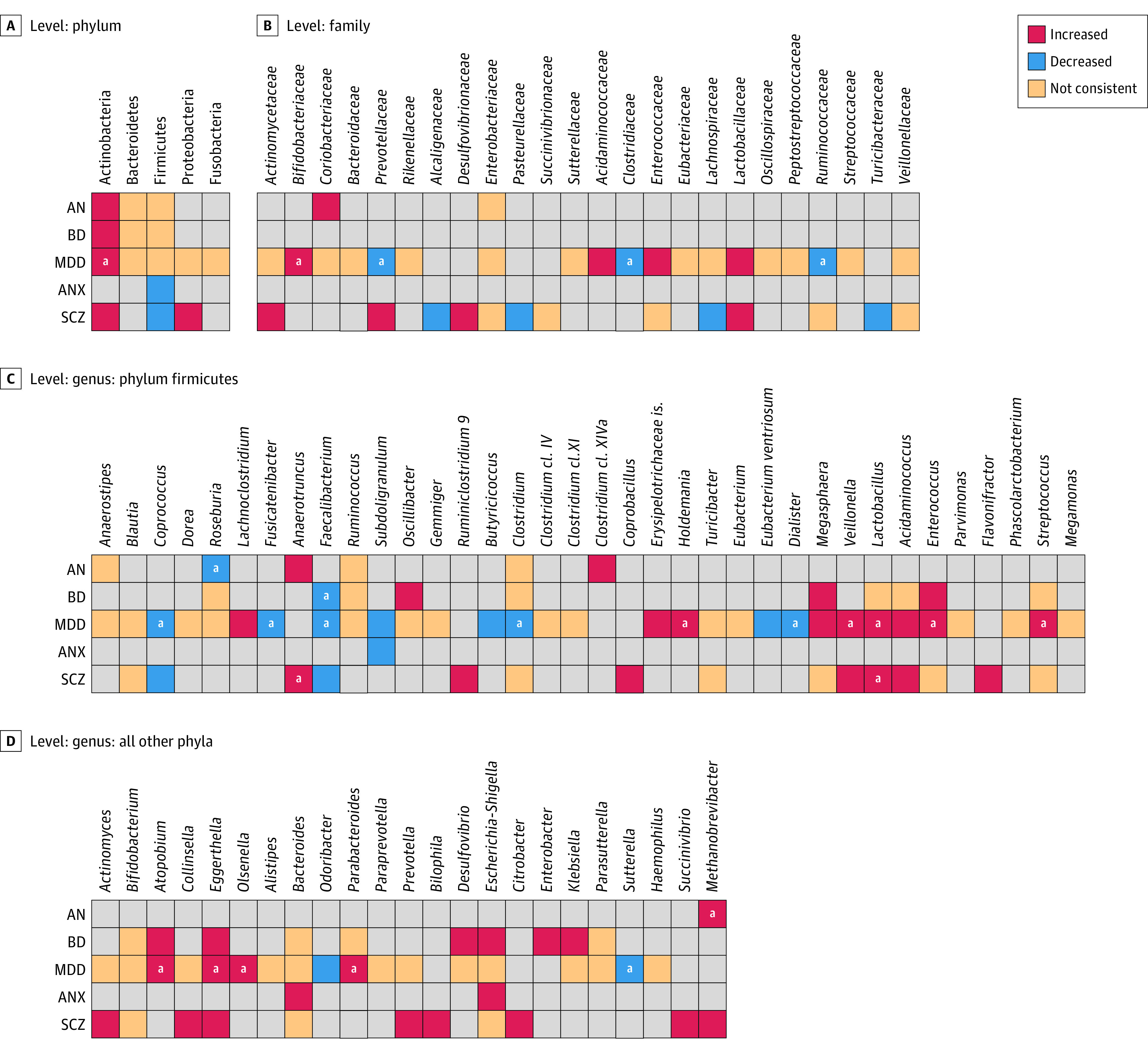
Changes in Relative Abundance of Microbial Taxa Reported by at Least 2 Studies From a Diagnostic Category Gray cells indicate not examined, not reported, or not replicated. ^a^Most replicated findings are indicated here, all of which have been reported by more than 1 research group. Number of studies: anorexia nervosa (AN), 10; bipolar disorder (BD), 9; major depressive disorder (MDD), 21; anxiety (ANX), 2; psychosis and schizophrenia (SCZ), 11.

Figure 3 provides the summary of the within- and between-disorder comparison for the disorders with sufficient studies (anorexia nervosa, MDD, bipolar disorder, anxiety, and psychosis and schizophrenia). There was high within-disorder inconsistency and the majority of consistent within-disorder changes were replicated by only 2 studies and thus require further investigation. Considerably fewer were replicated by more than 2 studies from different research groups.

#### Limited Evidence of Disorder Specificity

Disorder specificity was observed for the enrichment of genera *Holdemania* and *Olsenella* and the depletion of genera *Fusicatenibacter, Dialister,* and *Sutterella* in MDD (Figure 3C). However, these findings were weakly reproduced (3 to 4 of 21 studies). The archaeon *Methanobrevibacter* and genus *Anaerotruncus* may also be candidates for disorder specificity because they were consistently associated with anorexia nervosa and psychosis and schizophrenia, respectively. Interestingly, an alteration in the same direction was also reported in 2 studies from the other disorder, which could not be explained by apparent demographic, clinical, or methodological factors. Nevertheless, specificity in anorexia nervosa cannot be assessed here because no studies in other eating disorders were identified, and conditions such as obesity were beyond the scope. No distinct disorder-specific alterations were observed for the remaining taxa.

#### Transdiagnostic Alterations

Our findings indicate an overlap between certain disorders: bipolar disorder, psychosis and schizophrenia, and anxiety were associated with MDD. The most consistent changes were depletion of *Faecalibacterium* (in 15 of 17 studies reporting this genus) and *Coprococcus* (10 of 10 studies) and the enrichment of *Eggerthella* (in 10 of 11 studies) (eAppendix 10 in the Supplement). These were followed by enriched *Lactobacillus* (10 of 13 studies), *Enterococcus* (8 of 9 studies), and *Streptococcus* (8 of 10 studies). Further, *Atopobium* was enriched in bipolar disorder and MDD (5 of 5 studies), while *Veillonella* was enriched in psychosis and schizophrenia and MDD (5 of 6 studies). There was also evidence for the increase of the pathogen *Escherichia-Shigella *in bipolar disorder, anxiety, and psychosis and schizophrenia (6 of 7 studies) but not MDD. The *Bifidobacterium* and *Bacteroides *genera were reported frequently but inconsistently across these disorders (14 and 16 studies, respectively).

#### Exploring Confounders: Region and Psychiatric Medication

We explored the association of study region (east/west) with microbial alterations. Owing to the limited overlap in findings and the imbalanced availability of studies by region (eg, MDD and psychosis and schizophrenia were largely investigated in the east, while anorexia nervosa and OCD were investigated in the west), this analysis should be considered preliminary. Clustering according to region identified several taxa that were altered only in studies from Eastern countries: *Acidaminococcus* (increased), *Blautia* (not consistent), *Megamonas* (decreased), *Megasphaera* (increased), *Atopobium* (increased), and *Bacteroides* (not consistent). These differences were driven entirely by studies from China, highlighting the need to distinguish the Chinese microbiome from other East Asian nations as more evidence becomes available.

There is evidence that psychiatric medication can affect microbiota composition.^16,54^ To investigate this, we compared results from medication-free studies (n = 11) with those in which 80% or more of patients were taking medication (n = 21). We found that increases in the family *Lactobacillaceae* (although not member genus *Lactobacillus)* and the genera *Clostridium*, *Klebsiella,* and *Megasphaera* were only reported in medicated groups, while *Dialister* was decreased in medicated and increased in medication-free groups. Further, 6 of 8 studies in treated patients reported increases in *Streptococcus*, which was not reported in drug-free studies.

## Discussion

To our knowledge, this is the first review to assess gut microbiota perturbations across a spectrum of psychiatric disorders with the aim of evaluating the reproducibility and specificity of potential gut microbial biomarkers. The pattern of alterations observed suggests an increased magnitude and complexity of microbial disorganization for some disorders compared with others. For example, the highest number of differentially abundant taxa was in psychosis and schizophrenia (136 taxa; 11 studies), despite almost twice as many studies in MDD (94 taxa; 21 studies). Conversely, anorexia nervosa was associated with fewer differences (32 taxa; 10 studies), despite the larger number of studies compared with anxiety (36 taxa; 2 studies) and bipolar disorder (60 taxa; 9 studies). This is reminiscent of genome-wide association studies’ findings, in which the highest number of loci have been associated with psychosis and schizophrenia followed by MDD and bipolar disorder, and fewer have been associated with anorexia nervosa, PTSD, and ADHD.^55^ This increased complexity, also reflected in the microbiota, is consistent with the wider spectrum of clinical presentations associated with the former compared with the latter set of disorders.

Overall, we did not find evidence for disorder specificity: whenever microbial alterations merited specificity, these were weakly reproduced, suggesting they may instead reflect specific population characteristics (eg, depression subtype) and thus need further verification. Instead, our findings indicated that certain disorders share similar patterns of microbial changes. Specifically, we observed an overlap between psychosis and schizophrenia, bipolar disorder, anxiety, and MDD in consistently and inconsistently altered taxa, suggesting these likely harbor transdiagnostic alterations associated with overlapping pathophysiology as has previously been seen in analyses of inflammatory markers, neutrophil-lymphocyte ratios, and genome-wide association studies.^12,56,57^

Most consistently, the genus *Eggerthella* was enriched in MDD, bipolar disorder, and psychosis and schizophrenia, while the genera *Faecalibacterium *and *Coprococcus *were decreased in all. *Eggerthella* is associated with gastrointestinal inflammation,^10,58^ while *Faecalibacterium* has known anti-inflammatory properties^59^ and is depleted in immune-mediated inflammatory diseases.^58,60^ These associations are likely mediated by short-chain fatty acid butyrate, as *Faecalibacterium* and *Coprococcus* are involved in its production,^61^ while *Eggerthella* has been associated with its depletion.^10^ Butyrate has a key role in maintaining mucosal integrity and reducing inflammation via macrophage function and decrease in proinflammatory cytokines, while increasing anti-inflammatory mediators.^61,62,63^ Further, *Faecalibacterium* was inversely associated with depression severity in 2 MDD studies, 1 bipolar disorder study, and 1 anorexia nervosa study,^28,43,64,65^ suggesting depletion of this genus may be characteristic of the depressive state, irrespective of diagnosis. Therefore, clinical features and underlying pathophysiology that manifest across diagnoses may be better suited to explain the observed microbial alterations than distinct diagnostic categories. The merits of incorporating the gut microbiota as a dimensional component to the Research Domain Criteria^66^ have previously been discussed^67^ and our results reinforce this by demonstrating that while gut microbiota abnormalities were ubiquitously observed, these do not seem to congregate according to distinct diagnoses but instead exhibit a transdiagnostic pattern.

Interestingly, the family *Lactobacillaceae* and member genus *Lactobacillus*, strains from which are components of probiotic supplements and linked to positive health outcomes,^68^ were enriched in MDD, psychosis and schizophrenia, and bipolar disorder. A possible explanation could be that species from this genus have differential effect. For example, 1 study identified the increase in psychosis and schizophrenia to be in subspecies not typically present in the healthy gut.^53^ Alternatively, increased *Lactobacillus* has previously been associated with antipsychotic use.^69^ This finding was somewhat corroborated here, as 4 psychosis and schizophrenia studies reporting increased *Lactobacillus* were conducted in medicated groups,^32,34,46,70^ while the one that reported decreased *Lactobacillus* was in a treatment-naive group.^71^ In our exploratory analyses, the family *Lactobacillaceae* was significantly increased only in medicated groups. This suggests that psychotropic medication may be exacerbating the presence of illness-associated *Lactobacilli* species.

Measures of alpha diversity (within sample) were widely used, following the general assumption that higher diversity is more beneficial to the host^72^ and thus expected to be decreased in psychiatric patients, as has previously been observed for various diseases.^73^ However, our meta-analysis demonstrated a nonsignificant association with diversity indices and small to medium decrease in richness, suggesting that while richness is somewhat compromised (although the clinical significance of this decrease is unclear), diversity is overall preserved. The high residual heterogeneity following subgrouping according to disorder type suggests that diagnosis is not a good discriminator of alpha diversity. Regarding beta diversity (between samples), patients with MDD and psychosis and schizophrenia consistently clustered differently from controls. However, it is yet unknown whether psychiatric disorders cluster differently from one another, thus questioning the suitability of diversity measures as biomarkers. From the studies summarized, only 2 studies compared beta diversity cross-diagnostically and neither found a significant difference.^21,25^

Among the numerous clinical and demographic factors that may have contributed to the widespread inconsistencies between studies, current evidence allowed us to explore 2 key characteristics: geographical region and psychiatric medication. Geographical region and the associated factor of diet can profoundly affect the composition of the microbiota.^74,75^ Our analysis suggested that some of the observed perturbations may be specific to Chinese populations (eg, increased *Acidaminococcus*), others may be owing to the effect of psychiatric medication (eg, increased *Klebsiella* and decreased Dialister), while third may be influenced by a combination of both, such as the genus *Megasphaera*, which was enriched only in Chinese populations undergoing treatment. Future studies should be encouraged to report findings (even nonsignificant) on all dominant taxa to help delineate the effect of confounders from true disease effects. Additionally, more studies will be needed in currently underrepresented populations from low- to middle-income countries, as mental health problems become an increasing concern.^76^

For brevity, we have not discussed methodological differences that may have contributed to inconsistent findings such as processing, sequencing, or analysis pipelines because these have been extensively reviewed by others.^9,10,74,77,78^ Further, some have suggested that the current approaches to microbiome analyses may be unreliable owing to inappropriate handling of inherently compositional data.^79^ The lack of power calculations is a significant deterrent in the field. To move forward, the reporting of quantitative effect sizes of abundance findings in addition to *P* values is needed to enable meta-analyses and the evaluation of potentially relevant biological effects.^80^ Even then, technical and clinical variation between studies may make it difficult to compare effect sizes, which reinforces the need of harmonizing methodologies and encouraging data sharing with sufficient metadata.

### Limitations

Although there were insufficient studies to perform in-depth analyses of OCD, PTSD, and anxiety, we believe the inclusion of these disorders provides a comprehensive overview of current evidence. There were no studies in adults with autism spectrum disorder and only 1 study in ADHD, thus precluding us from comparing the association of neurodevelopmental disorders with the microbiota in adulthood. The decision to exclude studies in children and elderly individuals was dictated by an appreciation of the specialist nature of these populations and the substantial age-related differences in the microbiota.^81^ Next, we acknowledge that the division into Eastern and Western countries is a crude approach to controlling for geographical differences in diet and genetics and does not allow detection of regional variations in the microbiome, which might also explain why we found no alterations specific to Western populations. As more studies become available, more nuanced analyses will be possible. Additionally, most studies had modest sample sizes, suggesting our analyses may still be underpowered and preliminary. Similarly, as most studies included both medicated and unmedicated patients, our analyses of the confounding effects of medication require further verification in larger stratified populations. Our summary may also suffer from the use of different reference databases between studies, as inconsistencies in assigning taxonomy have been described.^82^ Finally, the aim of this review was to evaluate gut microbial composition, rather than function. Early evidence has suggested that functional potentials associated with psychiatric illness include short-chain fatty acid synthesis, tryptophan metabolism, and neurotransmitter synthesis/degradation.^52,53,83,84^ Given the noted functional redundancy,^85^ functional analysis will be key in understanding the role of host-microbiome interactions in neuropsychiatric disorders.

## Conclusions

This review suggests a transdiagnostic commonality of microbial disturbances in MDD, bipolar disorder, anxiety, and psychosis and schizophrenia, characterized by depleted anti-inflammatory butyrate-producing bacteria and enriched proinflammatory bacteria. The effect of key confounders such as psychiatric medication and diet should be carefully considered. Researchers should interpret their findings within the larger context of psychiatric disorders to prevent unmerited claims of disorder specificity of gut microbial biomarkers. The evidence summarized here is a good starting point for such comparisons.

## Notes

Supplement. eAppendix 1. Systematic search details eAppendix 2. Quality assessment eAppendix 3. Detailed methods of the meta-analysis performed eAppendix 4. PRISMA flowcharts for the umbrella review search and the updated review searches eAppendix 5. Details of the identified systematic reviews eAppendix 6. Detailed characteristics of the included studies eAppendix 7. Stool sample processing methods in the included studies eAppendix 8. Publication bias assessment for the alpha diversity meta-analyses eAppendix 9. Beta diversity eAppendix 10. Figures for study-level findings of relative abundance of microbial taxa
