# Supplementary material for: Perturbations in Gut Microbiota Composition in Psychiatric Disorders: A Review and Meta-analysis
Source: JAMA Psychiatry. 2021 Sep 15;78(12):1–12. doi: 10.1001/jamapsychiatry.2021.2573 (PMC8444066; doi:10.1001/jamapsychiatry.2021.2573)
Supplement: Supplement. — eAppendix 1. Systematic search details eAppendix 2. Quality assessment eAppendix 3. Detailed methods of the meta-analysis performed eAppendix 4. PRISMA flowcharts for the umbrella review search and the updated review searches eAppendix 5. Details of the identified systematic reviews eAppendix 6. Detailed characteristics of the included studies eAppendix 7. Stool sample processing methods in the included studies eAppendix 8. Publication bias assessment for the alpha diversity meta-analyses eAppendix 9. Beta diversity eAppendix 10. Figures for study-level findings of relative abundance of microbial taxa [file jamapsychiatry-e212573-s001.pdf]

Supplemental Online Content

Nikolova VL, Smith MRB, Hall LJ, Cleare AJ, Stone JM, Young AH. Perturbations in gut microbiota composition in psychiatric disorders: a systematic review and meta-analysis. *JAMA Psychiatry*. Published online September 15, 2021. doi:10.1001/jamapsychiatry.2021.2573

eAppendix 1. Systematic search details

eAppendix 2. Quality assessment

eAppendix 3. Detailed methods of the meta-analysis performed

eAppendix 4. PRISMA flowcharts for the umbrella review search and the updated review searches

eAppendix 5. Details of the identified systematic reviews

eAppendix 6. Detailed characteristics of the included studies

eAppendix 7. Stool sample processing methods in the included studies

eAppendix 8. Publication bias assessment for the alpha diversity meta-analyses

eAppendix 9. Beta diversity

eAppendix 10. Figures for study-level findings of relative abundance of microbial taxa

This supplemental material has been provided by the authors to give readers additional information about their work.

© 2021 American Medical Association. All rights reserved.

eAppendix 1. Systematic search details

1. Systematic reviews and meta-analysis search – performed on 27 Jan 2021

1.1. Cochrane Library

Search	Hits
Cochrane Reviews	8498
Topic Mental Health	637
AND gut OR gastrointestinal OR intestinal OR feacal OR fecal OR stool	56
AND microbiome OR microbiota OR ecosystem OR bacteria OR flora OR microflora OR dysbiosis	0

1.2. PubMed*

Search	Hits
((gut) OR (gastrointestinal) OR (intestinal) OR (feacal) OR (fecal) OR (stool))<br>AND ((microbiome) OR (microbiota) OR (ecosystem) OR (bacteria) OR (flora) OR (microflora) OR (dysbiosis))<br>AND ((depression) OR (depress*) OR (mdd) OR (trd) OR (bipolar) OR (mania) OR (bipolar depression) OR (anxiety) OR (psychosis) OR (schizophrenia) OR (obsessive compulsive disorder) OR (ocd) OR (ptsd) OR (post-traumatic stress disorder) OR (adhd) OR (attention deficit hyperactivity disorder) OR (autism) OR (autism spectrum disorder) OR (ASD) OR (eating disorder) OR (anorexia) OR (bulimia))	3699
AND (systematicreview[Filter])	80
Limits: 2005-current	79
Limits: human	47
Limits: English	**45**

1.3. Embase & PsychINFO (via Ovid)

Search	Hits
1. gut.mp. [mp=ti, ab, hw, tn, ot, dm, mf, dv, kw, fx, dq, tc, id, tm, mh]	138475
2. gastrointestinal.mp. [mp=ti, ab, hw, tn, ot, dm, mf, dv, kw, fx, dq, tc, id, tm, mh]	612345
3. intestinal.mp. [mp=ti, ab, hw, tn, ot, dm, mf, dv, kw, fx, dq, tc, id, tm, mh]	370239
4. feacal.mp. [mp=ti, ab, hw, tn, ot, dm, mf, dv, kw, fx, dq, tc, id, tm, mh]	167
5. fecal.mp. [mp=ti, ab, hw, tn, ot, dm, mf, dv, kw, fx, dq, tc, id, tm, mh]	87064
6. stool.mp. [mp=ti, ab, hw, tn, ot, dm, mf, dv, kw, fx, dq, tc, id, tm, mh]	60712
7. microbiome.mp. [mp=ti, ab, hw, tn, ot, dm, mf, dv, kw, fx, dq, tc, id, tm, mh]	42743
8. microbiota.mp. [mp=ti, ab, hw, tn, ot, dm, mf, dv, kw, fx, dq, tc, id, tm, mh]	68233
9. ecosystem.mp. [mp=ti, ab, hw, tn, ot, dm, mf, dv, kw, fx, dq, tc, id, tm, mh]	111701
10. bacteria.mp. [mp=ti, ab, hw, tn, ot, dm, mf, dv, kw, fx, dq, tc, id, tm, mh]	464415
11. flora.mp. [mp=ti, ab, hw, tn, ot, dm, mf, dv, kw, fx, dq, tc, id, tm, mh]	108206
12. microflora.mp. [mp=ti, ab, hw, tn, ot, dm, mf, dv, kw, fx, dq, tc, id, tm, mh]	56856
13. dysbiosis.mp. [mp=ti, ab, hw, tn, ot, dm, mf, dv, kw, fx, dq, tc, id, tm, mh]	14943

© 2021 American Medical Association. All rights reserved.

14. depression.mp. [mp=ti, ab, hw, tn, ot, dm, mf, dv, kw, fx, dq, tc, id, tm, mh]	1041348
15. depress*.mp. [mp=ti, ab, hw, tn, ot, dm, mf, dv, kw, fx, dq, tc, id, tm, mh]	1193818
16. MDD.mp. [mp=ti, ab, hw, tn, ot, dm, mf, dv, kw, fx, dq, tc, id, tm, mh]	32383
17. TRD.mp. [mp=ti, ab, hw, tn, ot, dm, mf, dv, kw, fx, dq, tc, id, tm, mh]	3628
18. bipolar.mp. [mp=ti, ab, hw, tn, ot, dm, mf, dv, kw, fx, dq, tc, id, tm, mh]	159279
19. mania.mp. [mp=ti, ab, hw, tn, ot, dm, mf, dv, kw, fx, dq, tc, id, tm, mh]	42988
20. bipolar depression.mp. [mp=ti, ab, hw, tn, ot, dm, mf, dv, kw, fx, dq, tc, id, tm, mh]	10336
21. anxiety.mp. [mp=ti, ab, hw, tn, ot, dm, mf, dv, kw, fx, dq, tc, id, tm, mh]	626469
22. psychosis.mp. [mp=ti, ab, hw, tn, ot, dm, mf, dv, kw, fx, dq, tc, id, tm, mh]	186882
23. schizophrenia.mp. [mp=ti, ab, hw, tn, ot, dm, mf, dv, kw, fx, dq, tc, id, tm, mh]	341812
24. obsessive compulsive disorder.mp. [mp=ti, ab, hw, tn, ot, dm, mf, dv, kw, fx, dq, tc, id, tm, mh]	50116
25. OCD.mp. [mp=ti, ab, hw, tn, ot, dm, mf, dv, kw, fx, dq, tc, id, tm, mh]	26104
26. PTSD.mp. [mp=ti, ab, hw, tn, ot, dm, mf, dv, kw, fx, dq, tc, id, tm, mh]	72988
27. post-traumatic stress disorder.mp. [mp=ti, ab, hw, tn, ot, dm, mf, dv, kw, fx, dq, tc, id, tm, mh]	28176
28. autism.mp. [mp=ti, ab, hw, tn, ot, dm, mf, dv, kw, fx, dq, tc, id, tm, mh]	134153
29. Autism spectrum disorder.mp. [mp=ti, ab, hw, tn, ot, dm, mf, dv, kw, fx, dq, tc, id, tm, mh]	42153
30. pervasive developmental disorder.mp. [mp=ti, ab, hw, tn, ot, dm, mf, dv, kw, fx, dq, tc, id, tm, mh]	4354
31. attention deficit hyperactivity disorder.mp. [mp=ti, ab, hw, tn, ot, dm, mf, dv, kw, fx, dq, tc, id, tm, mh]	61349
32. ADHD.mp. [mp=ti, ab, hw, tn, ot, dm, mf, dv, kw, fx, dq, tc, id, tm, mh]	69132
33. eating disorder.mp. [mp=ti, ab, hw, tn, ot, dm, mf, dv, kw, fx, dq, tc, id, tm, mh]	49763
34. anorexia.mp. [mp=ti, ab, hw, tn, ot, dm, mf, dv, kw, fx, dq, tc, id, tm, mh]	108326
35. bulimia.mp. [mp=ti, ab, hw, tn, ot, dm, mf, dv, kw, fx, dq, tc, id, tm, mh]	28823
36. systematic review.mp. [mp=ti, ab, hw, tn, ot, dm, mf, dv, kw, fx, dq, tc, id, tm, mh]	386059
37. meta-analysis.mp. [mp=ti, ab, hw, tn, ot, dm, mf, dv, kw, fx, dq, tc, id, tm, mh]	338776
38. 1 or 2 or 3 or 4 or 5 or 6	1066368
39. 7 or 8 or 9 or 10 or 11 or 12 or 13	693756
40. 14 or 15 or 16 or 17 or 18 or 19 or 20 or 21 or 22 or 23 or 24 or 25 or 26 or 27 or 28 or 29 or 30 or 31 or 32 or 33 or 34 or 35	2248227
41. 36 or 37	557086
42. 38 AND 39 AND 40 AND 41	252
43. limit 42 to english language	241
44. limit 43 to human	236
45. limit 44 to yr="2005 -Current"	**234**

© 2021 American Medical Association. All rights reserved.

2. Supplementary searches – all performed on 02 Feb 2021

2.1. PTSD & OCD – no date restriction as no systematic reviews were available

2.1.1. PubMed

Search	Hits
((gut) OR (gastrointestinal) OR (intestinal) OR (feacal) OR (fecal) OR (stool))<br>AND ((microbiome) OR (microbiota) OR (ecosystem) OR (bacteria) OR (flora) OR (microflora) OR (dysbiosis))<br>AND ((obsessive compulsive disorder) OR (ocd) OR (ptsd) OR (post-traumatic stress disorder)	45
Limits: human, English	20

2.1.2. Embase & PsychINFO (via Ovid)

Search	Hits
1. gut.mp. [mp=ti, ab, hw, tn, ot, dm, mf, dv, kw, fx, dq, tc, id, tm, mh]	138693
2. gastrointestinal.mp. [mp=ti, ab, hw, tn, ot, dm, mf, dv, kw, fx, dq, tc, id, tm, mh]	612886
3. intestinal.mp. [mp=ti, ab, hw, tn, ot, dm, mf, dv, kw, fx, dq, tc, id, tm, mh]	370633
4. feacal.mp. [mp=ti, ab, hw, tn, ot, dm, mf, dv, kw, fx, dq, tc, id, tm, mh]	167
5. fecal.mp. [mp=ti, ab, hw, tn, ot, dm, mf, dv, kw, fx, dq, tc, id, tm, mh]	87197
6. stool.mp. [mp=ti, ab, hw, tn, ot, dm, mf, dv, kw, fx, dq, tc, id, tm, mh]	60767
7. microbiome.mp. [mp=ti, ab, hw, tn, ot, dm, mf, dv, kw, fx, dq, tc, id, tm, mh]	42848
8. microbiota.mp. [mp=ti, ab, hw, tn, ot, dm, mf, dv, kw, fx, dq, tc, id, tm, mh]	68365
9. ecosystem.mp. [mp=ti, ab, hw, tn, ot, dm, mf, dv, kw, fx, dq, tc, id, tm, mh]	111818
10. bacteria.mp. [mp=ti, ab, hw, tn, ot, dm, mf, dv, kw, fx, dq, tc, id, tm, mh]	464960
11. flora.mp. [mp=ti, ab, hw, tn, ot, dm, mf, dv, kw, fx, dq, tc, id, tm, mh]	108206
12. microflora.mp. [mp=ti, ab, hw, tn, ot, dm, mf, dv, kw, fx, dq, tc, id, tm, mh]	56939
13. dysbiosis.mp. [mp=ti, ab, hw, tn, ot, dm, mf, dv, kw, fx, dq, tc, id, tm, mh]	14988
14. obsessive compulsive disorder.mp. [mp=ti, ab, hw, tn, ot, dm, mf, dv, kw, fx, dq, tc, id, tm, mh]	50157
15. OCD.mp. [mp=ti, ab, hw, tn, ot, dm, mf, dv, kw, fx, dq, tc, id, tm, mh]	26128
16. PTSD.mp. [mp=ti, ab, hw, tn, ot, dm, mf, dv, kw, fx, dq, tc, id, tm, mh]	73062
17. post-traumatic stress disorder.mp. [mp=ti, ab, hw, tn, ot, dm, mf, dv, kw, fx, dq, tc, id, tm, mh]	28199
18. 1 or 2 or 3 or 4 or 5 or 6	1067443
19. 7 or 8 or 9 or 10 or 11 or 12 or 13	694634
20. 14 or 15 or 16 or 17	133639
21. 18 AND 19 AND 20	98
22. limit 42 to english language	95
23. limit 43 to human	66

2.2. Anxiety & Depression - last review search of both disorders done in March 2020 (Simpson et al., 2021)

2.2.1. Pubmed

© 2021 American Medical Association. All rights reserved.

Search	Hits
((gut) OR (gastrointestinal) OR (intestinal) OR (feacal) OR (fecal) OR (stool))<br><br>AND ((microbiome) OR (microbiota) OR (ecosystem) OR (bacteria) OR (flora) OR (microflora) OR (dysbiosis))<br><br>AND ((depression) OR (depress*) OR (mdd) OR (trd) OR (anxiety))<br><br>AND NOT (review)	1714
Limits: human, English, start date: February 2020	42

2.2.2. Embase & PsychINFO (via Ovid)

Search	Hits
1. gut.mp. [mp=ti, ab, hw, tn, ot, dm, mf, dv, kw, fx, dq, tc, id, tm, mh]	138693
2. gastrointestinal.mp. [mp=ti, ab, hw, tn, ot, dm, mf, dv, kw, fx, dq, tc, id, tm, mh]	612886
3. intestinal.mp. [mp=ti, ab, hw, tn, ot, dm, mf, dv, kw, fx, dq, tc, id, tm, mh]	370633
4. feacal.mp. [mp=ti, ab, hw, tn, ot, dm, mf, dv, kw, fx, dq, tc, id, tm, mh]	167
5. fecal.mp. [mp=ti, ab, hw, tn, ot, dm, mf, dv, kw, fx, dq, tc, id, tm, mh]	87197
6. stool.mp. [mp=ti, ab, hw, tn, ot, dm, mf, dv, kw, fx, dq, tc, id, tm, mh]	60767
7. microbiome.mp. [mp=ti, ab, hw, tn, ot, dm, mf, dv, kw, fx, dq, tc, id, tm, mh]	42848
8. microbiota.mp. [mp=ti, ab, hw, tn, ot, dm, mf, dv, kw, fx, dq, tc, id, tm, mh]	68365
9. ecosystem.mp. [mp=ti, ab, hw, tn, ot, dm, mf, dv, kw, fx, dq, tc, id, tm, mh]	111818
10. bacteria.mp. [mp=ti, ab, hw, tn, ot, dm, mf, dv, kw, fx, dq, tc, id, tm, mh]	464960
11. flora.mp. [mp=ti, ab, hw, tn, ot, dm, mf, dv, kw, fx, dq, tc, id, tm, mh]	108206
12. microflora.mp. [mp=ti, ab, hw, tn, ot, dm, mf, dv, kw, fx, dq, tc, id, tm, mh]	56939
13. dysbiosis.mp. [mp=ti, ab, hw, tn, ot, dm, mf, dv, kw, fx, dq, tc, id, tm, mh]	14988
14. depression.mp. [mp=ti, ab, hw, tn, ot, dm, mf, dv, kw, fx, dq, tc, id, tm, mh]	1047721
15. depress*.mp. [mp=ti, ab, hw, tn, ot, dm, mf, dv, kw, fx, dq, tc, id, tm, mh]	1200820
16. MDD.mp. [mp=ti, ab, hw, tn, ot, dm, mf, dv, kw, fx, dq, tc, id, tm, mh]	32605
17. TRD.mp. [mp=ti, ab, hw, tn, ot, dm, mf, dv, kw, fx, dq, tc, id, tm, mh]	3663
18. anxiety.mp. [mp=ti, ab, hw, tn, ot, dm, mf, dv, kw, fx, dq, tc, id, tm, mh]	630521
19. 1 or 2 or 3 or 4 or 5 or 6	1067443
20. 7 or 8 or 9 or 10 or 11 or 12 or 13	694634
21. 14 or 15 or 16 or 17 or 18	1511690
22. 19 AND 20 AND 21	2774
23. limit 22 to english language	2687
24. limit 23 to human	1894
25. limit 24 to yr="2020 -Current"	509
26. review.mp. [mp=ti, ab, hw, tn, ot, dm, mf, dv, kw, fx, dq, tc, id, tm, mh]	4370959
27. 25 NOT 26	278

© 2021 American Medical Association. All rights reserved.

2.3. Bipolar Disorder & Psychosis/Schizophrenia - last review search of both disorders done on 17 Jan 2019 (Vindegaard et al., 2020)

2.3.1. PubMed

Search	Hits
((gut) OR (gastrointestinal) OR (intestinal) OR (feacal) OR (fecal) OR (stool))<br>AND ((microbiome) OR (microbiota) OR (ecosystem) OR (bacteria) OR (flora) OR (microflora) OR (dysbiosis))<br>AND ((bipolar) OR (mania) OR (bipolar depression) OR (psychosis) OR (schizophrenia))<br>AND NOT (review)	199
Limits: human, English, start date: January 2019	84

2.3.2. Embase & PsychINFO (via Ovid)

Search	Hits
1. gut.mp. [mp=ti, ab, hw, tn, ot, dm, mf, dv, kw, fx, dq, tc, id, tm, mh]	138693
2. gastrointestinal.mp. [mp=ti, ab, hw, tn, ot, dm, mf, dv, kw, fx, dq, tc, id, tm, mh]	612886
3. intestinal.mp. [mp=ti, ab, hw, tn, ot, dm, mf, dv, kw, fx, dq, tc, id, tm, mh]	370633
4. feacal.mp. [mp=ti, ab, hw, tn, ot, dm, mf, dv, kw, fx, dq, tc, id, tm, mh]	167
5. fecal.mp. [mp=ti, ab, hw, tn, ot, dm, mf, dv, kw, fx, dq, tc, id, tm, mh]	87197
6. stool.mp. [mp=ti, ab, hw, tn, ot, dm, mf, dv, kw, fx, dq, tc, id, tm, mh]	60767
7. microbiome.mp. [mp=ti, ab, hw, tn, ot, dm, mf, dv, kw, fx, dq, tc, id, tm, mh]	42848
8. microbiota.mp. [mp=ti, ab, hw, tn, ot, dm, mf, dv, kw, fx, dq, tc, id, tm, mh]	68365
9. ecosystem.mp. [mp=ti, ab, hw, tn, ot, dm, mf, dv, kw, fx, dq, tc, id, tm, mh]	111818
10. bacteria.mp. [mp=ti, ab, hw, tn, ot, dm, mf, dv, kw, fx, dq, tc, id, tm, mh]	464960
11. flora.mp. [mp=ti, ab, hw, tn, ot, dm, mf, dv, kw, fx, dq, tc, id, tm, mh]	108206
12. microflora.mp. [mp=ti, ab, hw, tn, ot, dm, mf, dv, kw, fx, dq, tc, id, tm, mh]	56939
13. dysbiosis.mp. [mp=ti, ab, hw, tn, ot, dm, mf, dv, kw, fx, dq, tc, id, tm, mh]	14988
14. bipolar.mp. [mp=ti, ab, hw, tn, ot, dm, mf, dv, kw, fx, dq, tc, id, tm, mh]	160089
15. mania.mp. [mp=ti, ab, hw, tn, ot, dm, mf, dv, kw, fx, dq, tc, id, tm, mh]	43186
16. bipolar depression.mp. [mp=ti, ab, hw, tn, ot, dm, mf, dv, kw, fx, dq, tc, id, tm, mh]	10387
17. psychosis.mp. [mp=ti, ab, hw, tn, ot, dm, mf, dv, kw, fx, dq, tc, id, tm, mh]	187899
18. schizophrenia.mp. [mp=ti, ab, hw, tn, ot, dm, mf, dv, kw, fx, dq, tc, id, tm, mh]	343421
19. 1 or 2 or 3 or 4 or 5 or 6	1067443
20. 7 or 8 or 9 or 10 or 11 or 12 or 13	694634
21. 14 or 15 or 16 or 17 or 18	577348
22. 19 AND 20 AND 21	661
23. limit 22 to 6English language	631
24. limit 23 to human	551
25. limit 24 to yr="2019 -Current"	291
26. review.mp. [mp=ti, ab, hw, tn, ot, dm, mf, dv, kw, fx, dq, tc, id, tm, mh]	4370959

© 2021 American Medical Association. All rights reserved.

27. 25 NOT 26	148

2.4. ADHD & ASD: last review search of both disorders done on 31Aug2018 (Jurek et al., 2020).

2.4.1. **PubMed**

Search	Hits
((gut) OR (gastrointestinal) OR (intestinal) OR (feacal) OR (fecal) OR (stool))<br>AND ((microbiome) OR (microbiota) OR (ecosystem) OR (bacteria) OR (flora) OR (microflora) OR (dysbiosis))<br>AND ((adhd) OR (attention deficit hyperactivity disorder) OR (autism) OR (autism spectrum disorder) OR (ASD))<br>AND NOT (review)	415
Limits: human, English, start date: August 2018	20

2.4.2. Embase & PsychINFO (via Ovid)

Search	Hits
1. gut.mp. [mp=ti, ab, hw, tn, ot, dm, mf, dv, kw, fx, dq, tc, id, tm, mh]	138693
2. gastrointestinal.mp. [mp=ti, ab, hw, tn, ot, dm, mf, dv, kw, fx, dq, tc, id, tm, mh]	612886
3. intestinal.mp. [mp=ti, ab, hw, tn, ot, dm, mf, dv, kw, fx, dq, tc, id, tm, mh]	370633
4. feacal.mp. [mp=ti, ab, hw, tn, ot, dm, mf, dv, kw, fx, dq, tc, id, tm, mh]	167
5. fecal.mp. [mp=ti, ab, hw, tn, ot, dm, mf, dv, kw, fx, dq, tc, id, tm, mh]	87197
6. stool.mp. [mp=ti, ab, hw, tn, ot, dm, mf, dv, kw, fx, dq, tc, id, tm, mh]	60767
7. microbiome.mp. [mp=ti, ab, hw, tn, ot, dm, mf, dv, kw, fx, dq, tc, id, tm, mh]	42848
8. microbiota.mp. [mp=ti, ab, hw, tn, ot, dm, mf, dv, kw, fx, dq, tc, id, tm, mh]	68365
9. ecosystem.mp. [mp=ti, ab, hw, tn, ot, dm, mf, dv, kw, fx, dq, tc, id, tm, mh]	111818
10. bacteria.mp. [mp=ti, ab, hw, tn, ot, dm, mf, dv, kw, fx, dq, tc, id, tm, mh]	464960
11. flora.mp. [mp=ti, ab, hw, tn, ot, dm, mf, dv, kw, fx, dq, tc, id, tm, mh]	108206
12. microflora.mp. [mp=ti, ab, hw, tn, ot, dm, mf, dv, kw, fx, dq, tc, id, tm, mh]	56939
13. dysbiosis.mp. [mp=ti, ab, hw, tn, ot, dm, mf, dv, kw, fx, dq, tc, id, tm, mh]	14988
14. attention deficit hyperactivity disorder.mp. [mp=ti, ab, hw, tn, ot, dm, mf, dv, kw, fx, dq, tc, id, tm, mh]	61699
15. adhd.mp. [mp=ti, ab, hw, tn, ot, dm, mf, dv, kw, fx, dq, tc, id, tm, mh]	69524
16. autism.mp. [mp=ti, ab, hw, tn, ot, dm, mf, dv, kw, fx, dq, tc, id, tm, mh]	135286
17. autism spectrum disorder.mp. [mp=ti, ab, hw, tn, ot, dm, mf, dv, kw, fx, dq, tc, id, tm, mh]	42650
18. ASD.mp. [mp=ti, ab, hw, tn, ot, dm, mf, dv, kw, fx, dq, tc, id, tm, mh]	56686
19. 1 or 2 or 3 or 4 or 5 or 6	1067443
20. 7 or 8 or 9 or 10 or 11 or 12 or 13	694634
21. 14 or 15 or 16 or 17 or 18	221434
22. 19 AND 20 AND 21	1209
23. limit 22 to english language	1159

© 2021 American Medical Association. All rights reserved.

24. limit 23 to human	962
25. limit 24 to yr="2018 -Current"	531
26. adult. mp. [mp=ti, ab, hw, tn, ot, dm, mf, dv, kw, fx, dq, tc, id, tm, mh]	8881500
27. 25 AND 26	63
28. review.mp. [mp=ti, ab, hw, tn, ot, dm, mf, dv, kw, fx, dq, tc, id, tm, mh]	4370959
29. 27 NOT 28	46

2.5. Eating disorders: last review search done in June 2020 (Di Ludovico et al., 2021).

2.5.1. **PubMed**

Search	Hits
((gut) OR (gastrointestinal) OR (intestinal) OR (feacal) OR (fecal) OR (stool))<br>AND ((microbiome) OR (microbiota) OR (ecosystem) OR (bacteria) OR (flora) OR (microflora) OR (dysbiosis))<br>AND ((eating disorder) OR (anorexia) OR (bulimia))<br>AND NOT (review)	489
Limits: human, English, start date: May 2020	13

2.5.2. Embase & PsychINFO (via Ovid)

Search	Hits
30. gut.mp. [mp=ti, ab, hw, tn, ot, dm, mf, dv, kw, fx, dq, tc, id, tm, mh]	138693
31. gastrointestinal.mp. [mp=ti, ab, hw, tn, ot, dm, mf, dv, kw, fx, dq, tc, id, tm, mh]	612886
32. intestinal.mp. [mp=ti, ab, hw, tn, ot, dm, mf, dv, kw, fx, dq, tc, id, tm, mh]	370633
33. feacal.mp. [mp=ti, ab, hw, tn, ot, dm, mf, dv, kw, fx, dq, tc, id, tm, mh]	167
34. fecal.mp. [mp=ti, ab, hw, tn, ot, dm, mf, dv, kw, fx, dq, tc, id, tm, mh]	87197
35. stool.mp. [mp=ti, ab, hw, tn, ot, dm, mf, dv, kw, fx, dq, tc, id, tm, mh]	60767
36. microbiome.mp. [mp=ti, ab, hw, tn, ot, dm, mf, dv, kw, fx, dq, tc, id, tm, mh]	42848
37. microbiota.mp. [mp=ti, ab, hw, tn, ot, dm, mf, dv, kw, fx, dq, tc, id, tm, mh]	68365
38. ecosystem.mp. [mp=ti, ab, hw, tn, ot, dm, mf, dv, kw, fx, dq, tc, id, tm, mh]	111818
39. bacteria.mp. [mp=ti, ab, hw, tn, ot, dm, mf, dv, kw, fx, dq, tc, id, tm, mh]	464960
40. flora.mp. [mp=ti, ab, hw, tn, ot, dm, mf, dv, kw, fx, dq, tc, id, tm, mh]	108206
41. microflora.mp. [mp=ti, ab, hw, tn, ot, dm, mf, dv, kw, fx, dq, tc, id, tm, mh]	56939
42. dysbiosis.mp. [mp=ti, ab, hw, tn, ot, dm, mf, dv, kw, fx, dq, tc, id, tm, mh]	14988
43. eating disorder.mp. [mp=ti, ab, hw, tn, ot, dm, mf, dv, kw, fx, dq, tc, id, tm, mh]	50029
44. anorexia.mp. [mp=ti, ab, hw, tn, ot, dm, mf, dv, kw, fx, dq, tc, id, tm, mh]	108993
45. bulimia.mp. [mp=ti, ab, hw, tn, ot, dm, mf, dv, kw, fx, dq, tc, id, tm, mh]	28918
46. 1 or 2 or 3 or 4 or 5 or 6	1067443
47. 7 or 8 or 9 or 10 or 11 or 12 or 13	694634
48. 14 or 15 or 16	152111

© 2021 American Medical Association. All rights reserved.

49. 17 AND 18 AND 19	550
50. limit 20 to english language	512
51. limit 21 to human	392
52. limit 22 to yr="2020 -Current"	83
53. review.mp. [mp=ti, ab, hw, tn, ot, dm, mf, dv, kw, fx, dq, tc, id, tm, mh]	4370959
54. 23 NOT 24	37

© 2021 American Medical Association. All rights reserved.

eAppendix 2. Quality assessment

Two authors (VLN and MRBS) performed the rating independently and resolved discrepancies via discussion.

Summary: The overall risk of bias in the systematic reviews was low (12/16) (Table S5.1), however, only 3/16 had a pre-registered protocol (Table S3.1). The most frequent concerns were the lack of clarity how evidence was collected and evaluated for quality and the thoroughness of the search strategy (Figure S5.1). However, due to the significant overlap, a recent high-quality review was available for each disorder. Regarding original studies published after the reviews, quality was high as assessed with the JBI tool for case-control studies. The primary concern was incomplete consideration of confounders with 7/20 either not identifying these and/or not accounting for them in the analyses. We did not penalize studies that considered only some factors (e.g. age, gender, psychiatric medication) as other, particularly lifestyle factors such as diet, are difficult to fully control.

Table e2.1. Quality assessment of the included systematic reviews using the ROBIS tool.

Review			Phase 1				Phase 2
**Disorder**	**First Author**	**Year**	**1. Study eligibility criteria**	**2. Identification and selection of studies**	**3. Data collection and study appraisal**	**4. Synthesis and findings**	**RISK OF BIAS IN THE REVIEW***
AN	Di Lodovico	2021	low	low	low	low	low
AN	Schalla	2019	low	high	unclear	high	low
AN	Schwensen	2018	unclear	high	low	low	low
MDD, ANX	Simpson	2021	low	low	low	low	low
MDD, SCZ	Fond	2020	high	high	unclear	unclear	high
MDD	Li	2020	unclear	high	unclear	high	high
MDD, ANX	Simpson	2020	low	low	low	low	low
MDD	Sanada	2020	low	low	low	low	low
MDD, BD, SCZ	Vindegaard	2020	low	low	low	low	low
MDD	Cheung	2019	low	high	high	unclear	unclear
MDD	Barandouzi	2020	low	low	low	high	low
BD, SCZ	Nguyen	2019	low	low	unclear	low	low
BD, SCZ	Nguyen	2018	high	unclear	unclear	low	low
SCZ	Cuomo	2018	high	high	high	high	high
SCZ	Kraeuter	2020	low	low	low	low	low
ASD, ADHD	Jurek	2020	low	low	low	low	low

Low = low risk of bias, high = high risk of bias, unclear = insufficient information to assess risk of bias; *this criterion is scored by answering the following questions: A. Did the interpretation of findings address all of the concerns identified the Phase 2 assessment?; B. Was the relevance of identified studies to the review's research question appropriately considered?; and C. Did the reviewers avoid emphasizing results on the basis of their statistical significance?.

© 2021 American Medical Association. All rights reserved.

Table e2.2. Quality assessment of the included original studies published after the systematic reviews using the Joanna Briggs Institute Critical Appraisal Checklist for Case Control Studies.

**Study** (First Author & Year)	**1. Were the criteria for inclusion in the sample clearly defined?**	**2. Were the study subjects and the setting described in detail?**	**3. Was the exposure measured in a valid and reliable way?**	**4. Were objective, standard criteria used for measurement of the condition?**	**5. Were confounding factors identified?**	**6. Were strategies to deal with confounding factors stated?**	**7. Were the outcomes measured in a valid and reliable way?**	**8. Was appropriate statistical analysis used?**
Chen 2020	yes	yes	yes	yes	yes	yes	yes	yes
Chen 2021	yes	yes	yes	yes	yes	yes	yes	yes
Yang 2020	yes	yes	yes	yes	yes	yes	yes	yes
Liu 2020	yes	yes	yes	yes	yes	yes	yes	yes
Stevens 2020	no	no	yes	yes	no	no	yes	yes
Jiang 2020	yes	yes	yes	yes	yes	no	yes	yes
McIntyre 2019	yes	yes	yes	yes	no	no	yes	yes
Hu 2019	yes	yes	yes	yes	yes	yes	yes	yes
Lai 2021	yes	yes	yes	yes	yes	yes	yes	yes
Lu 2019	yes	yes	yes	yes	yes	yes	yes	unclear
Pan 2020	yes	yes	yes	yes	yes	no	yes	yes
Zhu 2020	yes	yes	yes	yes	yes	yes	yes	yes
Li 2020	yes	yes	yes	yes	yes	no	yes	yes
Ma 2020	yes	yes	yes	yes	yes	no	yes	yes
Zhang 2020	yes	yes	yes	yes	yes	no	yes	yes
Xu 2020	yes	yes	yes	yes	yes	yes	yes	yes
Monteleone 2020	yes	yes	yes	yes	yes	yes	yes	yes
Hemmings 2017	yes	yes	yes	yes	yes	yes	yes	yes
Domenech n/a	yes	yes	yes	yes	yes	yes	yes	yes
Turna 2020	yes	yes	yes	yes	yes	yes	yes	yes

© 2021 American Medical Association. All rights reserved.

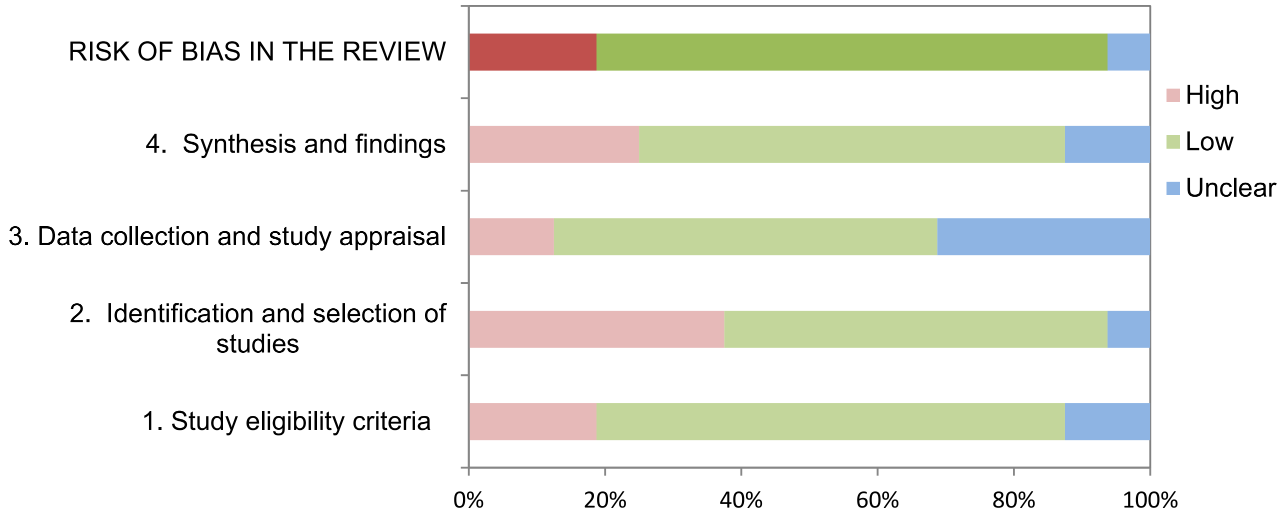

Figure e5.1. Quality assessment of the included systematic reviews using the ROBIS tool.

© 2021 American Medical Association. All rights reserved.

eAppendix 3: Detailed methods of the meta-analysis performed

Medians and inter-quartile ranges were transformed to means (M) and standard deviations (SD) using a web-based tool (http://www.math.hkbu.edu.hk/~tongt/papers/median2mean.html). For significantly skewed data, an alternative validated procedure was followed[1]. Where necessary, numerical data were extracted from graphs using WebPlotDigitizer (v.4.4[2]) and Adobe Acrobat's inbuilt measuring tool (Adobe Systems, California, USA), as previously done by others[3]. A random-effects meta-analysis on Hedge's g standardised mean difference (SMD) was performed applying the inverse-variance method. Effect size was categorized as small (SMD≤0.2), moderate (SMD=0.5), or large (SMD=0.8). Inter-study heterogeneity was quantified using the DerSimonian–Laird estimator, reported with the I2 statistic and interpreted according to convention (25% - low, 50% - moderate, and 75% - high)[4]. Publication bias was evaluated with funnel plots and Egger's regression test. Pre-planned subgroup analyses were disorder, region of study (east/west) and use of psychiatric medication. All analyses were completed with the meta package (v.4.17-0[5]) in R.

References:

1. Wan X, Wang W, Liu J, Tong T. Estimating the sample mean and standard deviation from the sample size, median, range and/or interquartile range. BMC Med Res Methodol. 2014;14. doi:10.1186/1471-2288-14-135

2. Rohatgi A. WebPlotDigitizer.; 2020. https://automeris.io/WebPlotDigitizer

3. Safadi JM, Quinton AMG, Lennox BR, Burnet PWJ, Minichino A. Gut dysbiosis in severe mental illness and chronic fatigue: a novel trans-diagnostic construct? A systematic review and meta-analysis. Molecular Psychiatry. Published online February 8, 2021:1-13. doi:10.1038/s41380-021-01032-1

4. Higgins DJPT. Cochrane Handbook for Systematic Reviews of Interventions. John Wiley & Sons; 2008. Accessed December 17, 2020.

5. Balduzzi S, Rücker G, Schwarzer G. How to perform a meta-analysis with R: a practical tutorial. Evid Based Ment Health. 2019;22(4):153-160. doi:10.1136/ebmental-2019-300117

© 2021 American Medical Association. All rights reserved.

eAppendix 4: PRISMA flowcharts for the umbrella review search and the updated review searches

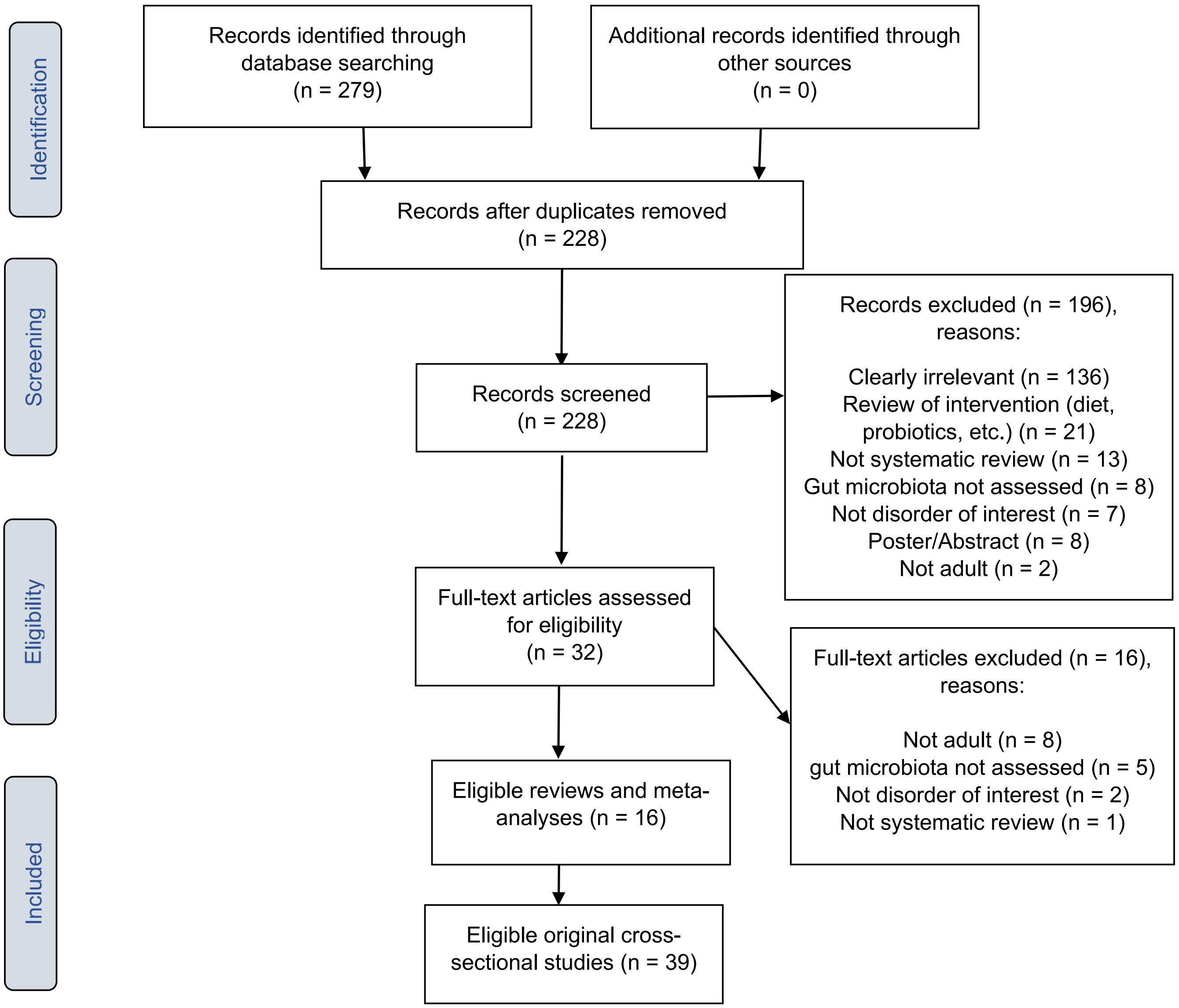

Figure e4.1. PRISMA flowchart of the umbrella review search

© 2021 American Medical Association. All rights reserved.

Table e4.1. PRISMA charts for the updated systematic review searches.

	**OCD & PTSD**	**MDD & ANX**	**BD & SCZ**	**ASD & ADHD**	**Eating disorders**
**Records identified**	86	293	209	66	50
**Reviewed**	86	293	209	66	50
**Excluded at Title/Abstract**	83	282	193	62	47
**Excluded at full text (with reasons)**	0	5 (n=1 gut microbiota not assessed; n=2 outcome of interest not reported; n=2 no control group)	6 (n=2 gut microbiota not assessed, n=1 outcome of interest not assessed; n=3 conference abstract)	4 (n=2 non-adult, n=1 non-human, n=1 gut microbiota not assessed)	2 (n=1 secondary analysis from included study, n=1 gut microbiota not analysed)
**Included**	**3 (2 OCD, 1 PTSD)**	**6 (5 MDD, 1 MDD + BD)**	**10 (6 SCZ, 4 BD)**	**0**	**1 (AN)**

© 2021 American Medical Association. All rights reserved.

eAppendix 5. Details of the identified systematic reviews

Table e5.1. Details of the identified systematic reviews.

First Author	Year	Ref.	Disorder	Studies included (eligible only)*	Pre-registered?
Di Lodovico	2021	[1]	anorexia	N = 9<br>Armougoum 2009; Million 2013; Kleiman 2015; Morita 2015; Mack 2016; Mörkl 2017; Borgo 2017; Hanachi 2018; Hata 2019	no
Schalla	2019	[2]	anorexia	N = 5<br>Morita et al., 2015; Mörkl et al., 2017; Kleiman et al., 2015; Borgo et al., 2017; Mack et al., 2016	no
Schwensen	2018	[3]	anorexia	N = 7<br>Mörkl et al., 2017; Borgo et al 2017; Kleiman et al 2015; Morita et al 2015; Amougom et al 2009; Million et al 2013; Mack et al 2016	Yes
Simpson	2021	[4]	depression	N = 18<br>Aizawa et al (2016); Chahwan et al. (2019); Chen, Li et al. (2018); Chen, Zheng et al. (2018); Chen et al. (2019); Chung et al. (2019); Huang et al. (2018); Jiang et al. (2015); Kelly et al. (2016); Lai et al., (2019); Lin et al. (2017); Liu et al. (2016); Mason et al., (2020); Naseribafrouei et al. (2014); Rong et al. (2019); Valles- Colomer et al. (2019); Vinberg et al. (2019); Zheng et al. (2016)	no
			anxiety	N = 3<br>Mason et al., (2020); Jiang et al. (2018); Chen et al. (2019)	
Fond	2020	[5]	depression	N = 7<br>Chen et al. 2018; Kelly et al. 2016; Liu et al. 2016; Jiang et al. 2015; Madan et al. 2020; Mason et al. 2020; Naseribafrouei et al., 2014	no
			schizophrenia	N = 0	
Li	2020	[6]	depression	N= 9<br>Naseribafrouei et al., 2014; Jiang et al., 2015; Kelly et al., 2016; Zheng et al., 2016; Huang et al., 2018; Chen et al., 2018b; Chung et al., 2019; Chen et al., 2018a; Rong et al., 2019	no
Simpson	2020	[7]	depression	N = 2<br>Liu et al. (2016), Aizawa et al. (2016)	no
Sanada	2020	[8]	depression	N = 10<br>Chen 2018a; Chen 2018b; Huang 2018; Lin 2017; Aizawa 2016; Kelly 2016; Liu 2016; Zheng 2016; Jiang 2015; Naseribafrouei 2014	no

© 2021 American Medical Association. All rights reserved.

Vindegaard	2020	[9]	depression	N = 9<br>Chen et al., 2018; Huang et al., 2018; Stevens et al., 2018; Lin et al., 2017; Aizawa et al., 2016; Kelly et al., 2016; Zheng et al., 2016; Jiang et al., 2015; Naseribafrouei et al., 2014	yes
			bipolar disorder	N = 4<br>Coello et al., 2019; Aizawa et al., 2018; Painold et al., 2018; Evans et al., 2017	
			psychosis	N = 4<br>Nguyen et al., 2018; Schwarz et al., 2018; Shen et al., 2018; Yuan et al., 2018	
Cheung	2019	[10]	depression	N = 6<br>Naseribafrouei et al 2014; Jiang et al 2015; Aizawa et al 2016; Zheng et al 2016; Lin et al 2017; Chen et al 2018	no
Barandouzi	2020	[11]	depression	N = 9<br>Chen et al 2018; Zheng et al 2016; Liu et al 2016; Chen et al 2018; Jiang et al 2015; Naserbafrouei et al 2014; Lin et al 2017; Aizawa et al 2016; Kelly et al 2016;	no
Nguyen	2019	[12]	bipolar disorder	N = 4<br>Painold et al., 2019; Evans et al 2017; Coello et al., 2019; Aizawa et al., 2018	no
			psychosis	N = 5<br>Schwarz et al., 2018; Yuan et al., 2018; Nagamine et al 2018; Nguyen et al 2019; Shen et al 2018	
Nguyen	2018	[13]	bipolar disorder	N = 1<br>Evans et al 2017	no
			psychosis	N = 1<br>Schwarz et al. (2017)	
Cuomo	2018	[14]	psychosis	N = 3<br>Schwarz et al. (2018); Shen et al (2018); Yuan et al (2018)	no
Kraeuter	2020	[15]	psychosis	N = 5<br>Schwarz et al 2018; Shen et al 2018; Yuan et al 2018; Nguyen et al 2018; Zheng 2019	no
Jurek	2020	[16]	autism, ADHD	N = 1<br>Aarts et al. 2017	yes

*after duplicate studies from these systematic reviews were removed, the total number of studies added through this route was 39.

eReferences:

1. Di Lodovico L, Mondot S, Doré J, Mack I, Hanachi M, Gorwood P. Anorexia nervosa and gut microbiota: A systematic review and quantitative synthesis of pooled microbiological data. Prog Neuropsychopharmacol Biol Psychiatry. 2020 Sep 22;110114.

2. Schalla MA, Stengel A. Gastrointestinal alterations in anorexia nervosa - A systematic review. Eur Eat Disord Rev. 2019;27(5):447–61.

3. Schwensen HF, Kan C, Treasure J, Høiby N, Sjögren M. A systematic review of studies on the faecal microbiota in anorexia nervosa: future research may need to include microbiota from the small intestine. Eat Weight Disord. 2018 Aug;23(4):399–418.

© 2021 American Medical Association. All rights reserved.

4. Simpson CA, Mu A, Haslam N, Schwartz OS, Simmons JG. Feeling down? A systematic review of the gut microbiota in anxiety/depression and irritable bowel syndrome. Journal of Affective Disorders [Internet]. 2020 Apr [cited 2020 Jun 2];266:429–46. Available from: https://linkinghub.elsevier.com/retrieve/pii/S0165032719325777

5. Fond GB, Lagier JC, Honore S, Lancon C, Korchia T, De Verville PLS, Llorca PM, Auquier P, Guedj E, Boyer L. Microbiota-orientated treatments for major depression and schizophrenia. Nutrients [Internet]. 2020;12(4).

6. Li S, Hua D, Wang Q, Yang L, Wang X, Luo A, Yang C. The Role of Bacteria and Its Derived Metabolites in Chronic Pain and Depression: Recent Findings and Research Progress. Int J Neuropsychopharmacol. 2020 10;23(1):26–41.

7. Simpson CA, Diaz-Arteche C, Eliby D, Schwartz OS, Simmons JG, Cowan CSM. The gut microbiota in anxiety and depression - A systematic review. Clin Psychol Rev. 2020 Oct 29;83:101943.

8. Sanada K, Nakajima S, Kurokawa S, Barceló-Soler A, Ikuse D, Hirata A, Yoshizawa A, Tomizawa Y, Salas-Valero M, Noda Y, Mimura M, Iwanami A, Kishimoto T. Gut microbiota and major depressive disorder: A systematic review and meta-analysis. J Affect Disord. 2020 01;266:1–13.

9. Vindegaard N, Speyer H, Nordentoft M, Rasmussen S, Benros ME. Gut microbial changes of patients with psychotic and affective disorders: A systematic review. Schizophr Res. 2020 Jan 14;

10. Cheung SG, Goldenthal AR, Uhlemann A-C, Mann JJ, Miller JM, Sublette ME. Systematic Review of Gut Microbiota and Major Depression. Front Psychiatry [Internet]. 2019 Feb 11 [cited 2020 Jun 2];10:34. Available from: https://www.frontiersin.org/article/10.3389/fpsyt.2019.00034/full

11. Barandouzi ZA, Starkweather AR, Henderson WA, Gyamfi A, Cong XS. Altered Composition of Gut Microbiota in Depression: A Systematic Review. Front Psychiatry. 2020;11:541.

12. Nguyen TT, Hathaway H, Kosciolek T, Knight R, Jeste DV. Gut microbiome in serious mental illnesses: A systematic review and critical evaluation. Schizophr Res. 2019 Sep 5;

13. Nguyen TT, Kosciolek T, Eyler LT, Knight R, Jeste DV. Overview and systematic review of studies of microbiome in schizophrenia and bipolar disorder. J Psychiatr Res. 2018;99:50–61.

14. Cuomo A, Maina G, Rosso G, Beccarini Crescenzi B, Bolognesi S, Di Muro A, Giordano N, Goracci A, Neal SM, Nitti M, Pieraccini F, Fagiolini A. The Microbiome: A New Target for Research and Treatment of Schizophrenia and its Resistant Presentations? A Systematic Literature Search and Review. Front Pharmacol [Internet]. 2018 Oct 15 [cited 2021 Mar 17];9. Available from: https://www.ncbi.nlm.nih.gov/pmc/articles/PMC6196757/

15. Kraeuter A-K, Phillips R, Sarnyai Z. The Gut Microbiome in Psychosis From Mice to Men: A Systematic Review of Preclinical and Clinical Studies. Front Psychiatry. 2020;11:799.

16. Jurek L, Sevil M, Jay A, Schröder C, Baghdadli A, Héry-Arnaud G, Geoffray M-M. Is there a dysbiosis in individuals with a neurodevelopmental disorder compared to controls over the course of development? A systematic review. Eur Child Adolesc Psychiatry. 2020 May 8;

© 2021 American Medical Association. All rights reserved.

eAppendix 6. Detailed characteristics of the included studies

The 59 studies provided 64 case-control comparisons capturing 2643 patients and 2336 controls (Table 6.1). Most studies (n=32) were conducted in East Asia (China, Taiwan or Japan), 24 in westernised populations (grouped on the basis of typical diet and lifestyle: USA, Canada, Europe, Australia, New Zealand) and one in Africa. All but one[12] used formal diagnostic criteria to define their population. Studies were similar in exclusion criteria such as major medical and gastrointestinal conditions, pregnancy and recent consumption of antibiotics (except four studies in which antibiotics weren't mentioned[17,25,28,33]). Recent probiotic consumption was excluded in 35/59 studies, was not mentioned in 21, and three studies included a small number of participants taking probiotics[3,27,50]. Few studies imposed restrictions on diet such as no major changes in the months preceding enrolment[10,19,35,36] or no weight loss, vegetarian or vegan diets[14,15,18,20,31]. Two studies matched groups according to diet[39,44] and one controlled dietary intake[55]. Despite its known impact on microbial communities[60], smoking was generally not controlled: only three studies excluded smokers[18,20,38] and five controlled for it during analyses[19,23,28,45,51]. Amplicon 16S rRNA sequencing was used in 44 studies, although choice of hypervariable region (V1-V9) varied, seven studies used shotgun metagenomics to sample all microbial genes, nine studies used either qPCR or RT-qPCR to target a pre-specified range of microbial taxa, and one study employed metaproteomic analysis (Table 6.1).

Supplementary Table 6.1. Key sample and methodology characteristics of case-control comparisons of the gut microbiome by disorder.

Disorder	Study	Country	Definition of disorder Stage	Sample size n	Mean Age	% Female	Mean BMI	% Smokers	% Patients on medication	Sequencing	Diversity assessments
**MDD**	Naseribafrouei et al. 2014 [1]	Norway	ICD-10	P: 37<br>HC: 18	P: 49.2<br>HC: 46.1	56%	P: 25.9<br>HC: 24.7	nr	Nr	16S rRNA<br>nr	α: Observed sp., Simpsons<br>β: not measured
**MDD**	Jiang et al. 2015 [2]	China	DSM-IV	P: 29<br>HC: 30	P: 25.3<br>HC: 26.8	38%<br>50%	P: 20.3<br>HC: 19.6	P: 10%<br>HC: 7%	most, total % nr	16S rRNA<br>V1-V3	α: Chao1, ACE, Shannon, Simpson, evenness; β: UniFrac (unweighted)
**MDD**	Aizawa et al. 2016 [3]	Japan	DSM-IV	P: 43<br>HC: 57	P: 39.4<br>HC: 42.8	41%<br>61%	P:23.2<br>HC: 22.3	nr	65%	RT-qPCR<br>16S rRNA	α: not measured<br>β: not measured
**MDD**	Kelly et al. 2016 [4]	Ireland	DSM-IV	P: 34<br>HC: 33	P: 45.8<br>HC: 45.8	38%	P: 26.2<br>HC: 24.6	P: 13%<br>HC: 3%	96%	16S rRNA<br>nr	α: Observed sp., Chao1, Shannon, PD<br>β: UniFrac (weighted & unweighted), Bray-Curtis
**MDD**	Liu et al. 2016 [5]	China	DSM-IV	P: 15<br>HC: 20	P: 44.8<br>HC: 43.9	69%	P: 22.0<br>HC: 22.0	nr	0%	16S rRNA<br>V1-V3	α: Observed sp., Chao1, Shannon, PD<br>β: measured, nr

© 2021 American Medical Association. All rights reserved.

Disorder	Study	Country	Definition of disorder Stage	Sample size n	Mean Age	% Female	Mean BMI	% Smokers	% Patients on medication	Sequencing	Diversity assessments
**MDD**	Zheng et al. 2016 [6]	China	DSM-IV	P: 58<br>HC: 63	P: 40.6<br>HC: 41.8	63%	P: 22.0<br>HC: 22.6	P: 16%<br>H: 21%	67%	16S rRNA<br>V3-V5	α: Observed sp., Shannon, Simpson, PD<br>β: Bray-Curtis
**MDD**	Lin et al. 2017 [7]	China	DSM-IV	P: 10<br>HC: 10	P: 36.2<br>HC: 38.1	60%	P: 23.8<br>HC: 24.2	P:40%<br>HC:30%	100%	16S rRNA<br>V3-V4	α: measured, nr<br>β: UniFrac (weighted)
**MDD**	Chen et al. 2018a [8]	China	HAMD	P: 44<br>HC: 44	P: 40.9<br>HC: 43.4	55%	P: 22.1<br>HC: 22.6	nr	0%<br>(drug-naïve)	16S rRNA seq<br>V3-V5	α: Observed sp., Shannon, Simpson, PD<br>β: UniFrac (nr), PLS-DA
**MDD**	Chen et al. 2018b [9]	China	DSM-IV	P: 10<br>HC: 10	P: 43.9<br>HC:39.6	50%	P: 23.5<br>HC: 22.6	nr	20%	Meta-proteomics	α: not measured<br>β: not measured
**MDD**	Huang et al. 2018 [10]	China	ICD-10	P: 27<br>HC: 27	P: 48.7<br>HC: 42.3	74%	P: 23.8<br>HC: 23.4	nr	Nr	16S rRNA<br>V3-V4	α: Chao1, ACE, Shannon, PD<br>β: UniFrac (weighted & unweighted)
**MDD**	Chahwan et al. 2019 [11]	Australia	M.I.N.I	P: 68<br>HC: 20	P: 36.1<br>HC: 40.0	70%	nr	P: 25%<br>HC: 11%	0%	16S rRNA<br>V3-V4	α: Observed sp., Chao1, Shannon<br>β: UniFrac (weighted)
**MDD**	Valles-Colomer 2019 [12]	Belgium / NL	GP & self-report	P: 80<br>HC: 70	50.9	55%	24.9	nr	50%	16S rRNA<br>nr	α: not measured<br>β: not measured
**MDD**	Chung et al. 2019 [13]	Taiwan	DSM-5	P: 36<br>HC: 37	P: 45.8<br>HC: 41.2	70%	P: 22.8<br>HC: 24	P: 19%<br>HC: 3%	86%	16S rRNA<br>V3-V4	α: Shannon<br>β: UniFrac (weighted)
**MDD**	Lai et al. 2019 [14]	China	DSM-5	P: 26<br>HC: 29	P: 43.7<br>HC: 39.4	P: 69%<br>HC: 55%	P: 27.2<br>HC: 21.1	nr	81%	Shotgun Metagenomics	α: Shannon, Fisher<br>β: Bray–Curtis
**MDD**	Rong et al. 2019 [15]	China	DSM-5	P: 31<br>HC: 30	P: 41.6<br>HC: 39.5	P: 71%<br>HC:53%	P: 21.5<br>HC: 22.0	nr	74%	Shotgun Metagenomics	α: Chao 1, Shannon, Inverse Simpson, Gm coefficient; β: Bray-Curtis
**MDD**	Mason et al. 2020 [16]	USA	DSM-IV	P: 14<br>HC: 10	P: 41.9<br>HC: 33.0	P:79%<br>HC: 60%	P: 31.0<br>HC: 25.6	nr	64%	16S rRNA<br>V4	α: Shannon<br>β: UniFrac (weighted)
**MDD**	Chen et al. 2020 [17]	China	DSM-IV	Young<br>P: 25<br>HC: 27<br>Mid-age<br>P: 45	Young<br>P: 24.0<br>HC: 25.0<br>Mid-age<br>P: 45.0	72%	Young P: 22.1<br>HC: 21.5<br>Mid-age<br>P:22.6	nr	Young: 28%<br>Mid-age: 31%	16SrRNA<br>V3-V5	α: Chao1, ACE<br>β: OPLS-DA

© 2021 American Medical Association. All rights reserved.

Disorder	Study	Country	Definition of disorder Stage	Sample size n	Mean Age	% Female	Mean BMI	% Smokers	% Patients on medication	Sequencing	Diversity assessments
				HC: 44	HC: 47.2		HC: 23.2				
**MDD**	Chen et al. 2021 [18]	China	DSM-5	P: 62<br>HC: 46	P: 39.6<br>HC: 34.0	100%	P: 22.0<br>HC: 22.2	0%	0%	16SrRNA V3-V4	α: Observed sp., Chao1, ACE, Shannon, Simpson;<br>β: UniFrac (weighted & unweighted)
**MDD**	Yang et al. 2020 [19]	China	DSM-IV	P: 156<br>HC: 155	P: 29.6<br>HC: 29.1	P: 36 %<br>HC: 54%	P: 22.3<br>HC: 22.4	nr	24%	Shotgun Metagenomics	α: Chao1, Shannon, Inv. Shannon<br>β: Bray-Curtis
**MDD**	Liu et al. 2020 [20]	USA	DSM-5	P: 43<br>HC: 47	P: 21.9<br>HC: 22.1	P: 88%<br>HC: 72%	nr	0%	65%	16SrRNA V4	α: Observed sp., Shannon, PD<br>β: UniFrac (weighted & unweighted), Bray-Curtis
**MDD**	Stevens et al. 2020 [21]	USA	DSM-IV	P: 20<br>HC: 20	P & HC: 34	P: 50%<br>HC: 70%	nr	nr	75%	nr	α: Chao1, Shannon<br>β: Bray-Curtis
**MDD + ANX**	Stevens et al. 2018 [22]	USA	DSM-5	P: 22<br>HC: 28	nr	nr	nr	nr	Nr	nr	α: not measured<br>β: not measured
**MDD & ANX**	Mason et al. 2020 [16]	USA	DSM-IV	P: 38<br>HC: 10	P:39.2<br>HC:33.0	P: 82%<br>HC: 60%	P: 20.4<br>HC: 25.6	nr	42%	16S rRNA V4	α: Shannon<br>β: UniFrac (weighted)
**MDD & BD**	Vinberg et al. 2019 [23]	Denmark	ICD-8 & ICD-10 remission	P: 74<br>HC: 25	P: 37.7<br>HC: 37.0	77%	P: 26.5<br>HC: 24.5	P > HC, % nr	61%	16SrRNA V3-V4	α: Observed sp., Shannon<br>β: generalized UniFrac
**MDD & BD**	Jiang et al. 2020 [24]	China	DSM-IV	MDD:14<br>BD: 10<br>HC: 16	P: 37.2<br>HC: 35.8	45%	P: 23.6<br>HC: 22.3	P: 8%<br>HC: 6%	most, total % nr	16SrRNA V1-V3	α: Chao1, ACE, Shannon, Simpson<br>β: UniFrac (weighted & unweighted), Bray-Curtis
**BD**	Evans et al. 2017 [25]	USA	DSM-IV	P:115<br>HC: 64	P: 50.2<br>HC: 48.6	73%	P: 29.3<br>HC: 26.0	nr	most, total % nr	16SrRNA V4	α: not measured<br>β: Yue & Clayton
**BD**	Painold et al. 2018 [26]	Austria	DSM-IV, current	P: 32<br>HC: 10	P: 41.3<br>HC: 31.4	44%	P: 28.4<br>HC: 24.3	nr	100%	16SrRNA V1-V2	α: Observed sp., Chao1, Shannon, Simpson<br>β: UniFrac (weighted & unweighted)

© 2021 American Medical Association. All rights reserved.

Disorder	Study	Country	Definition of disorder Stage	Sample size n	Mean Age	% Female	Mean BMI	% Smokers	% Patients on medication	Sequencing	Diversity assessments
			depression or euthymia								
**BD**	Aizawa et al. 2019 [27]	Japan	DSM-IV, any episode	P: 39<br>HC: 58	P: 40.3<br>HC: 43.1	56%	P: 23.9<br>HC: 22.4	nr	92%	RT qPCR for 16S/23S rRNA	α: not measured<br>β: not measured
**BD**	Rong et al. 2019 [15]	China	DSM-5, current depression	P: 30<br>HC: 30	P: 38.4<br>HC: 39.5	52%	P: 21.9<br>HC: 22.0	nr	83%	Shotgun Metagenomics	α: Chao 1, Shannon, Inv. Simpson, Gm coefficient<br>β: Bray-Curtis
**BD**	Coello et al. 2019 [28]	Denmark	ICD-10, any episode	P: 113<br>HC: 77	P: 31<br>HC: 29	62%	P: 24.8<br>HC:24.2	P: 36%<br>HC: 11%	88%	16SrRNA V3-V4	α: Observed sp., Shannon<br>β: UniFrac (weighted & unweighted)
**BD**	McIntyre et al. 2019 [29]	Canada	DSM-5, current depression	P: 23<br>HC: 23	P: 45<br>HC: 43.8	70%	P: 30 HC: 26	P: 21%<br>HC: 9%	nr	16SrRNA V3	α: Observed sp., Shannon, Inv. Simpson<br>β: Bray-Curtis
**BD**	Hu et al. 2019 [30]	China	DSM-IV-TR, current depression	P: 52<br>HC: 45	P: 24.2<br>HC:36.3	48%	P: 21.6<br>HC: 22.4	nr	0%	16SrRNA V3-V4	α: Observed sp., Chao1, Shannon, Simpson, Inv. Simpson, ICE<br>β: UniFrac (weighted & unweighted)
**BD**	Lai et al. 2021 [31]	China	DSM-5, current depression	P: 25<br>HC: 28	P: 36.9<br>HC: 39.2	48%	P: 22.1<br>HC:21.1	nr	80%	Shotgun Metagenomics	α: Shannon, Simpson, Fisher<br>β: Bray-Curtis
**BD**	Lu et al. 2019 [32]	China	DSM-IV-TR, current depression	P: 36<br>HC: 27	P: 32.6<br>HC: 28.9	43%	P: 22.2<br>HC: 21.8	0%	0%	qPCR	α: not measured<br>β: not measured
**SCZ**	Nguyen et al. 2019 [33]	USA	DSM-IV-TR	P: 25<br>HC: 25	P: 52.9<br>HC: 54.7	44%	P: 31.8<br>HC:28.9	P: 56%<br>HC: 4%	100%	16SrRNA V4	α: Observed sp., Shannon, Faith's PD<br>β: UniFrac (unweighted), Bray-Curtis
**SCZ**	Schwarz et al. 2018 [34]	Finland	DSM-IV; FEP	P: 28<br>HC: 16	P: 25.9<br>HC: 27.1	43%	P: 23.8<br>HC:23.9	nr	93%	qPCR for 16S rRNA primers, Metagenomics	α: not measured<br>β: not measured
**SCZ**	Shen et al. 2018 [35]	China	ICD-10	P: 64<br>HC: 53	P: 42<br>HC: 39	44%	P:23.5<br>HC:23.1	P: 19%<br>HC: 23%	100%	16SrRNA V3-V4	α: Observed sp., Chao1, ACE, Shannon, Simpson, Faith's PD<br>β: UniFrac (unweighted)

© 2021 American Medical Association. All rights reserved.

Disorder	Study	Country	Definition of disorder Stage	Sample size n	Mean Age	% Female	Mean BMI	% Smokers	% Patients on medication	Sequencing	Diversity assessments
**SCZ**	Yuan et al. 2018 [36]	China	DSM-IV; FEP	P: 41<br>HC: 41	P: 23.1<br>HC: 24.7	44%	P:20.5<br>HC: 20.8	P: 5%<br>HC: 6%	0%	qPCR for 16S rRNA primers	α: not measured<br>β: not measured
**SCZ**	Zheng et al. 2019 [37]	China	DSM-IV	P: 63<br>HC: 69	P: 43.5<br>HC: 40.0	P:33%<br>HC: 48%	P: 22.9<br>HC: 23.2	nr	92%	16SrRNA V3-V4	α: Chao 1, Shannon<br>β: PLS-DA
**SCZ**	Pan et al. 2020 [38]	China	DSM-IV	P: 29<br>HC: 29	P: 34.9<br>HC: 34.8	34%	P: 23.7<br>HC: 23.5	0%	90%	16SrRNA V3-V4	α: Observed sp., Chao1, ACE, Shannon, Simpson, Faith's PD<br>β: UniFrac (unweighted)
**SCZ**	Zhu et al. 2020 [39]	China	DSM-IV	P: 90<br>HC: 81	P: 28.6<br>HC: 32.3	49%	P: 20.6<br>HC: 21.7	P: 30%<br>HC: 25%	0%	Shotgun Metagenomics	α: Shannon<br>β: Bray-Curtis
**SCZ**	Li et al. 2020 [40]	China	DSM-IV-TR	P: 82<br>HC: 80	P: 42.2<br>HC: 41.0	47%	P:24.5<br>HC: 23.0	P:21%<br>HC: 5%	91%	16SrRNA V4	α: Observed Sp., Evenness, Shannon, Faith's PD;<br>β: Bray-Curtis
**SCZ**	Ma et al. 2020 [41]	China	DSM-IV; FEP & SCZ	FEP: 40<br>SCZ: 85<br>HC: 69	P: 24.2<br>HC:23.1	46%	nr	nr	FEP: 0% (drug-naïve)<br>SCZ: 100%	16SrRNA V4	α: Chao1, Shannon<br>β: UniFrac (weighted & unweighted)
**SCZ**	Zhang et al. 2020 [42]	China	DSM-IV; FEP	P:10<br>HC:16	P: 37.6<br>HC: 35.8	42%	P: 23.3<br>HC:22.3	P: 10%<br>HC: 6.3%	0% (drug naïve)	16SrRNA nr	α: Observed sp., Chao1, Shannon, Simpson; β: UniFrac (weighted & unweighted), Bray-Curtis
**SCZ**	Xu et al., 2020 [43]	China	DSM-5	P: 84<br>HC: 84	P: 35.0<br>HC: 35.0	43%	P:22<br>HC:23.1	nr	98%	16SrRNA V4 & Shotgun Metagenomics	α: Chao1<br>β: non-metric multidimensional scaling
**ANX**	Jiang et al. 2018 [44]	China	DSM-IV	P: 40<br>HC: 36	P: 33.4<br>HC: 35.6	P: 75%<br>HC: 64%	P:21.7<br>HC: 21.4	P: 2.5%<br>HC: 3%	70%	16SrRNA V3-V4	α: Observed sp., Chao1, ACE, Shannon, Simpson; β: UniFrac unweighted
**ANX**	Chen et al. 2019 [45]	China	DSM-5	P: 36<br>HC: 24	P: 46.1<br>HC: 41.8	57%	P: 23.1<br>HC: 22.5	P: 19.4%<br>HC: 16.7%	nr	16SrRNA V3-V4	α: Observed sp., Chao1, ACE, Shannon, Simpson<br>β: UniFrac (weighted & unweighted)

© 2021 American Medical Association. All rights reserved.

Disorder	Study	Country	Definition of disorder Stage	Sample size n	Mean Age	% Female	Mean BMI	% Smokers	% Patients on medication	Sequencing	Diversity assessments
**ANX**	Mason et al. 2020 [16]	USA	DSM-IV	P: 8<br>HC: 10	P: 40.0<br>HC: 33.0	P: 100%<br>HC: 60%	P: 33.3<br>HC: 25.6	nr	62%	16SrRNA V4	α: Shannon<br>β: UniFrac weighted
**AN**	Armougom et al. 2009 [46]	France	DSM-IV	P: 9<br>HC: 20	P: 19-36<br>HC: 13-68	nr	P: 12.7<br>HC: 20.7	nr	nr	RT qPCR	α: not measured<br>β: not measured
**AN**	Million et al. 2013 [47]	France	DSM-IV	P: 15<br>HC: 76	P: 27.3<br>HC: 49.5	P: 93%<br>HC:43 %	P: 13.5<br>HC: 22.4	nr	nr	qPCR	α: not measured<br>β: not measured
**AN**	Kleiman et al. 2015 [48]	USA	DSM-IV-TR	P: 16<br>HC: 12	P: 28.0<br>HC: 29.8	100%	P: 16.2<br>HC: 21.5	nr	nr	16SrRNA V1-V3	α: Observed sp., Chao1<br>β: UniFrac (weighted & unweighted)
**AN**	Morita et al. 2015 [49]	Japan	DSM-IV-TR	P: 25<br>HC: 21	P: 30.0<br>HC: 31.5	100%	P: 12.8<br>HC: 20.5	nr	nr	qPCR for 16S/23S rRNA	α: not measured<br>β: not measured
**AN**	Mack et al. 2016 [50]	Germany	'Diagnosis' (not specified)	P: 55<br>HC: 55	P: 23.8<br>HC: 23.7	100%	P: 15.3<br>HC: 21.6	nr	nr	16SrRNA V4	α: Observed sp., Chao 1, Shannon<br>β: UniFrac (weighted & unweighted), Bray-Curtis
**AN**	Mörkl et al. 2017 [51]	Austria	ICD-10	P: 18<br>HC: 26	P: 22.4<br>HC: 24.9	100%	P: 15.<br>HC: 21.9	25%	nr	16SrRNA V1-V2	α: Observed sp., Chao 1, Shannon<br>β: UniFrac (weighted & unweighted)
**AN**	Borgo et al. 2017 [52]	Italy	DSM-5	P: 15<br>HC: 15	P: 25.6<br>HC: 24.4	100%	P: 13.9<br>HC: 22.1	nr	nr	16SrRNA V3-V4	α: measured, nr<br>β: measured, nr
**AN**	Hanachi et al. 2018 [53]	France	DSM-IV-TR	P: 33<br>HC: 22	P: 32<br>HC: 36	100%	P: 11.7<br>HC: 21.0	nr	nr	16SrRNA V3-V4	α: Chao 1, Shannon<br>β: UniFrac (weighted & unweighted)
**AN**	Hata et al. 2019 [54]	Japan	DSM-IV-TR restrictive only	P: 4<br>HC: 4	P: 23.0<br>HC: 25.3	100%	P:13.7<br>HC:21.6	nr	nr	16SrRNA V3-V4	α: Observed sp., Chao 1, Shannon<br>β: UniFrac (weighted & unweighted)
**AN**	Monteleone et al. 2021 [55]	Italy	DSM-5	P:21<br>HC: 20	P: 21.7<br>HC:23.0	100%	P: 14.6<br>HC: 20.3	nr	nr	16SrRNA V4	α: Chao1, Fisher<br>β: non-metric multidimensional scaling
**OCD**	Domenech et al. *pre-print* [56]	Spain	DSM-IV	P: 38<br>HC: 33	P: 40.2<br>HC: 36.0	53%	nr	nr	nr	16SrRNA V3-V4	α: Observed sp., Chao 1, Shannon, Simpson, Inv. Simpson, Faith's PD<br>β: UniFrac (weighted & unweighted), Bray-Curtis, Jensen-Shannon, Canberra

© 2021 American Medical Association. All rights reserved.

Disorder	Study	Country	Definition of disorder Stage	Sample size n	Mean Age	% Female	Mean BMI	% Smokers	% Patients on medication	Sequencing	Diversity assessments
**OCD**	Turna et al. 2020 [57]	Canada	DSM-5	P: 21<br>HC: 22	P: 31.0<br>HC:29.3	54%	P: 24.6<br>HC: 23.2	nr	0%	16SrRNA V3	α: Observed sp., Chao 1, Inv. Simpson, Shannon;<br>β: UniFrac (weighted & unweighted), Bray-Curtis, Jaccard
**PTSD**	Hemmings et al. 2017 [58]	South Africa	DSM-5	P: 18<br>HC: 22	P: 42.0<br>HC: 38.7	P: 14%<br>HC: 7%	P:28.5<br>HC:28.6	P: 50%<br>HC:42%	33%	16SrRNA V3-V4	α: Observed sp., Chao1, Shannon, Faith's PD<br>β: UniFrac (weighted & unweighted), Bray Curtis
**ADHD**	Aarts et al. 2017 [59]	NL	DSM-IV	P: 19<br>HC:77	P: 19.5<br>HC: 27.1	P:32%<br>HC:47 %	P: 23.8<br>HC: 23.0	nr	nr	16SrRNA V3-V4	α: Observed sp., Chao1, Shannon, Faith's PD<br>β: not measured

ADHD attention deficit hyperactivity disorder, AN anorexia nervosa, ANX anxiety, BD bipolar disorder, OCD obsessive compulsive disorder, MDD major depressive disorder, PTSD post-traumatic stress disorder, SCZ schizophrenia and psychosis, BMI body mass index, P patient, HC healthy control, NL Netehrlands, ICD International Classification of Diseases, DSM Diagnostic and Statistical Manual of Mental Disorders, MINI Mini-International Neuropsychiatric Interview, FEP first episode psychosis, seq sequencing, (RT) qPCR (real time) quantitative polymerase chain reaction, ACE abundance-based coverage estimator, ICE incidence-based estimator, Faith's PD Faith's phylogenetic diversity, PLS-DA partial least squares discriminant analysis, OPLS-DA orthogonal projections to latent structures discriminant analysis, n number, nr not reported

eReferences:

1. Naseribafrouei A, Hestad K, Avershina E, et al. Correlation between the human fecal microbiota and depression. *Neurogastroenterol Motil Off J Eur Gastrointest Motil Soc*. 2014;26(8):1155-1162. doi:10.1111/nmo.12378

2. Jiang H, Ling Z, Zhang Y, et al. Altered fecal microbiota composition in patients with major depressive disorder. *Brain Behav Immun*. 2015;48:186-194. doi:10.1016/j.bbi.2015.03.016

3. Aizawa E, Tsuji H, Asahara T, et al. Possible association of Bifidobacterium and Lactobacillus in the gut microbiota of patients with major depressive disorder. *J Affect Disord*. 2016;202:254-257. doi:10.1016/j.jad.2016.05.038

4. Kelly JR, Borre Y, O' Brien C, et al. Transferring the blues: Depression-associated gut microbiota induces neurobehavioural changes in the rat. *J Psychiatr Res*. 2016;82:109-118. doi:10.1016/j.jpsychires.2016.07.019

© 2021 American Medical Association. All rights reserved.

5. Liu Y, Zhang L, Wang X, et al. Similar Fecal Microbiota Signatures in Patients With Diarrhea-Predominant Irritable Bowel Syndrome and Patients With Depression. *Clin Gastroenterol Hepatol*. 2016;14(11):1602-1611.e5. doi:10.1016/j.cgh.2016.05.033

6. Zheng P, Zeng B, Zhou C, et al. Gut microbiome remodeling induces depressive-like behaviors through a pathway mediated by the host's metabolism. *Mol Psychiatry*. 2016;21(6):786-796. doi:10.1038/mp.2016.44

7. Lin P, Ding B, Feng C, et al. Prevotella and Klebsiella proportions in fecal microbial communities are potential characteristic parameters for patients with major depressive disorder. *J Affect Disord*. 2017;207:300-304. doi:10.1016/j.jad.2016.09.051

8. Chen J, Zheng P, Liu Y, et al. Sex differences in gut microbiota in patients with major depressive disorder. *Neuropsychiatr Dis Treat*. 2018;14:647-655. doi:10.2147/NDT.S159322

9. Chen Z, Li J, Gui S, et al. Comparative metaproteomics analysis shows altered fecal microbiota signatures in patients with major depressive disorder. *Neuroreport*. 2018;29(5):417-425. doi:10.1097/WNR.0000000000000985

10. Huang Y, Shi X, Li Z, et al. Possible association of Firmicutes in the gut microbiota of patients with major depressive disorder. *Neuropsychiatr Dis Treat*. 2018;14:3329-3337. doi:10.2147/NDT.S188340

11. Chahwan B, Kwan S, Isik A, van Hemert S, Burke C, Roberts L. Gut feelings: A randomised, triple-blind, placebo-controlled trial of probiotics for depressive symptoms. *J Affect Disord*. 2019;253:317-326. doi:10.1016/j.jad.2019.04.097

12. Valles-Colomer M, Falony G, Darzi Y, et al. The neuroactive potential of the human gut microbiota in quality of life and depression. *Nat Microbiol*. 2019;4(4):623-632. doi:10.1038/s41564-018-0337-x

13. Chung Y-CE, Chen H-C, Chou H-CL, et al. Exploration of microbiota targets for major depressive disorder and mood related traits. *J Psychiatr Res*. 2019;111:74-82. doi:10.1016/j.jpsychires.2019.01.016

14. Lai W-T, Deng W-F, Xu S-X, et al. Shotgun metagenomics reveals both taxonomic and tryptophan pathway differences of gut microbiota in major depressive disorder patients. *Psychol Med*. Published online November 5, 2019:1-12. doi:10.1017/S0033291719003027

15. Rong H, Xie X, Zhao J, et al. Similarly in depression, nuances of gut microbiota: Evidences from a shotgun metagenomics sequencing study on major depressive disorder versus bipolar disorder with current major depressive episode patients. *J Psychiatr Res*. 2019;113:90-99. doi:10.1016/j.jpsychires.2019.03.017

© 2021 American Medical Association. All rights reserved.

16. Mason BL, Li Q, Minhajuddin A, et al. Reduced anti-inflammatory gut microbiota are associated with depression and anhedonia. *J Affect Disord*. 2020;266:394-401. doi:10.1016/j.jad.2020.01.137

17. Chen J-J, He S, Fang L, et al. Age-specific differential changes on gut microbiota composition in patients with major depressive disorder. *Aging*. 2020;12(3):2764-2776. doi:10.18632/aging.102775

18. Chen Y, Xue F, Yu S, et al. Gut microbiota dysbiosis in depressed women: The association of symptom severity and microbiota function. *J Affect Disord*. 2021;282:391-400. doi:10.1016/j.jad.2020.12.143

19. Yang J, Zheng P, Li Y, et al. Landscapes of bacterial and metabolic signatures and their interaction in major depressive disorders. *Sci Adv*. 2020;6(49):eaba8555. doi:10.1126/sciadv.aba8555

20. Liu RT, Rowan-Nash AD, Sheehan AE, et al. Reductions in anti-inflammatory gut bacteria are associated with depression in a sample of young adults. *Brain Behav Immun*. 2020;88:308-324. doi:10.1016/j.bbi.2020.03.026

21. Stevens BR, Roesch L, Thiago P, et al. Depression phenotype identified by using single nucleotide exact amplicon sequence variants of the human gut microbiome. *Mol Psychiatry*. Published online January 27, 2020:1-11. doi:10.1038/s41380-020-0652-5

22. Stevens BR, Goel R, Seungbum K, et al. Increased human intestinal barrier permeability plasma biomarkers zonulin and FABP2 correlated with plasma LPS and altered gut microbiome in anxiety or depression. *Gut*. 2018;67(8):1555-1557. doi:10.1136/gutjnl-2017-314759

23. Vinberg M, Ottesen NM, Meluken I, et al. Remitted affective disorders and high familial risk of affective disorders associate with aberrant intestinal microbiota. *Acta Psychiatr Scand*. 2019;139(2):174-184. doi:https://doi.org/10.1111/acps.12976

24. Jiang H, Pan L, Zhang X, Zhang Z, Zhou Y, Ruan B. Altered gut bacterial–fungal interkingdom networks in patients with current depressive episode. *Brain Behav*. 2020;10(8):e01677. doi:https://doi.org/10.1002/brb3.1677

25. Evans SJ, Bassis CM, Hein R, et al. The gut microbiome composition associates with bipolar disorder and illness severity. *J Psychiatr Res*. 2017;87:23-29. doi:10.1016/j.jpsychires.2016.12.007

26. Painold A, Mörkl S, Kashofer K, et al. A step ahead: Exploring the gut microbiota in inpatients with bipolar disorder during a depressive episode. *Bipolar Disord*. 2019;21(1):40-49. doi:10.1111/bdi.12682

27. Aizawa E, Tsuji H, Asahara T, et al. Bifidobacterium and Lactobacillus Counts in the Gut Microbiota of Patients With Bipolar Disorder and Healthy Controls. *Front Psychiatry*. 2019;9:730. doi:10.3389/fpsyt.2018.00730

© 2021 American Medical Association. All rights reserved.

28. Coello K, Hansen TH, Sørensen N, et al. Gut microbiota composition in patients with newly diagnosed bipolar disorder and their unaffected first-degree relatives. *Brain Behav Immun*. 2019;75:112-118. doi:10.1016/j.bbi.2018.09.026

29. McIntyre RS, Subramaniapillai M, Shekotikhina M, et al. Characterizing the gut microbiota in adults with bipolar disorder: a pilot study. *Nutr Neurosci*. Published online May 28, 2019:1-8. doi:10.1080/1028415X.2019.1612555

30. Hu S, Li A, Huang T, et al. Gut Microbiota Changes in Patients with Bipolar Depression. *Adv Sci*. 2019;6(14):1900752. doi:https://doi.org/10.1002/advs.201900752

31. Lai W, Zhao J, Xu S, et al. Shotgun metagenomics reveals both taxonomic and tryptophan pathway differences of gut microbiota in bipolar disorder with current major depressive episode patients. *J Affect Disord*. 2021;278:311-319. doi:10.1016/j.jad.2020.09.010

32. Lu Q, Lai J, Lu H, et al. Gut Microbiota in Bipolar Depression and Its Relationship to Brain Function: An Advanced Exploration. *Front Psychiatry*. 2019;10. doi:10.3389/fpsyt.2019.00784

33. Nguyen TT, Hathaway H, Kosciolek T, Knight R, Jeste DV. Gut microbiome in serious mental illnesses: A systematic review and critical evaluation. *Schizophr Res*. Published online September 5, 2019. doi:10.1016/j.schres.2019.08.026

34. Schwarz E, Maukonen J, Hyytiäinen T, et al. Analysis of microbiota in first episode psychosis identifies preliminary associations with symptom severity and treatment response. *Schizophr Res*. 2018;192:398-403. doi:10.1016/j.schres.2017.04.017

35. Shen Y, Xu J, Li Z, et al. Analysis of gut microbiota diversity and auxiliary diagnosis as a biomarker in patients with schizophrenia: A cross-sectional study. *Schizophr Res*. 2018;197:470-477. doi:10.1016/j.schres.2018.01.002

36. Yuan X, Zhang P, Wang Y, et al. Changes in metabolism and microbiota after 24-week risperidone treatment in drug naïve, normal weight patients with first episode schizophrenia. *Schizophr Res*. 2018;201:299-306. doi:10.1016/j.schres.2018.05.017

37. Zheng P, Zeng B, Liu M, et al. The gut microbiome from patients with schizophrenia modulates the glutamate-glutamine-GABA cycle and schizophrenia-relevant behaviors in mice. *Sci Adv*. 2019;5(2):eaau8317. doi:10.1126/sciadv.aau8317

38. Pan R, Zhang X, Gao J, Yi W, Wei Q, Su H. Analysis of the diversity of intestinal microbiome and its potential value as a biomarker in patients with schizophrenia: A cohort study. *Psychiatry Res*. 2020;291:113260. doi:10.1016/j.psychres.2020.113260

39. Zhu F, Ju Y, Wang W, et al. Metagenome-wide association of gut microbiome features for schizophrenia. *Nat Commun*. 2020;11(1):1612. doi:10.1038/s41467-020-15457-9

© 2021 American Medical Association. All rights reserved.

40. Li S, Zhuo M, Huang X, et al. Altered gut microbiota associated with symptom severity in schizophrenia. *PeerJ*. 2020;8. doi:10.7717/peerj.9574

41. Ma X, Asif H, Dai L, et al. Alteration of the gut microbiome in first-episode drug-naïve and chronic medicated schizophrenia correlate with regional brain volumes. *J Psychiatr Res*. 2020;123:136-144. doi:10.1016/j.jpsychires.2020.02.005

42. Zhang X, Pan L-Y, Zhang Z, Zhou Y-Y, Jiang H-Y, Ruan B. Analysis of gut mycobiota in first-episode, drug-naïve Chinese patients with schizophrenia: A pilot study. *Behav Brain Res*. 2020;379:112374. doi:10.1016/j.bbr.2019.112374

43. Xu R, Wu B, Liang J, et al. Altered gut microbiota and mucosal immunity in patients with schizophrenia. *Brain Behav Immun*. 2020;85:120-127. doi:10.1016/j.bbi.2019.06.039

44. Jiang H-Y, Zhang X, Yu Z-H, et al. Altered gut microbiota profile in patients with generalized anxiety disorder. *J Psychiatr Res*. 2018;104:130-136. doi:10.1016/j.jpsychires.2018.07.007

45. Chen Y-H, Bai J, Wu D, et al. Association between fecal microbiota and generalized anxiety disorder: Severity and early treatment response. *J Affect Disord*. 2019;259:56-66. doi:10.1016/j.jad.2019.08.014

46. Armougom F, Henry M, Vialettes B, Raccah D, Raoult D. Monitoring Bacterial Community of Human Gut Microbiota Reveals an Increase in Lactobacillus in Obese Patients and Methanogens in Anorexic Patients. *PLOS ONE*. 2009;4(9):e7125. doi:10.1371/journal.pone.0007125

47. Million M, Angelakis E, Maraninchi M, et al. Correlation between body mass index and gut concentrations of Lactobacillus reuteri , Bifidobacterium animalis , Methanobrevibacter smithii and Escherichia coli. *Int J Obes*. 2013;37(11):1460-1466. doi:10.1038/ijo.2013.20

48. Kleiman SC, Watson HJ, Bulik-Sullivan EC, et al. The Intestinal Microbiota in Acute Anorexia Nervosa and During Renourishment: Relationship to Depression, Anxiety, and Eating Disorder Psychopathology. *Psychosom Med*. 2015;77(9):969–981. doi:10.1097/PSY.0000000000000247

49. Morita C, Tsuji H, Hata T, et al. Gut Dysbiosis in Patients with Anorexia Nervosa. *PLOS ONE*. 2015;10(12):e0145274. doi:10.1371/journal.pone.0145274

50. Mack I, Cuntz U, Grämer C, et al. Weight gain in anorexia nervosa does not ameliorate the faecal microbiota, branched chain fatty acid profiles, and gastrointestinal complaints. *Sci Rep*. 2016;6:26752. doi:10.1038/srep26752

© 2021 American Medical Association. All rights reserved.

51. Mörkl S, Lackner S, Müller W, et al. Gut microbiota and body composition in anorexia nervosa inpatients in comparison to athletes, overweight, obese, and normal weight controls. *Int J Eat Disord*. 2017;50(12):1421-1431. doi:10.1002/eat.22801

52. Borgo F, Riva A, Benetti A, et al. Microbiota in anorexia nervosa: The triangle between bacterial species, metabolites and psychological tests. Sanz Y, ed. *PLOS ONE*. 2017;12(6):e0179739. doi:10.1371/journal.pone.0179739

53. Hanachi M, Manichanh C, Schoenenberger A, et al. Altered host-gut microbes symbiosis in severely malnourished anorexia nervosa (AN) patients undergoing enteral nutrition: An explicative factor of functional intestinal disorders? *Clin Nutr Edinb Scotl*. 2019;38(5):2304-2310. doi:10.1016/j.clnu.2018.10.004

54. Hata T, Miyata N, Takakura S, et al. The Gut Microbiome Derived From Anorexia Nervosa Patients Impairs Weight Gain and Behavioral Performance in Female Mice. *Endocrinology*. 2019;160(10):2441-2452. doi:10.1210/en.2019-00408

55. Monteleone AM, Troisi J, Fasano A, et al. Multi-omics data integration in anorexia nervosa patients before and after weight regain: A microbiome-metabolomics investigation. *Clin Nutr Edinb Scotl*. 2021;40(3):1137-1146. doi:10.1016/j.clnu.2020.07.021

56. Domènech L, Willis J, Alemany M, et al. Changes in the stool and oropharyngeal microbiome in obsessive-compulsive disorder. *medRxiv*. Published online May 28, 2020:2020.05.26.20113779. doi:10.1101/2020.05.26.20113779

57. Turna J, Grosman Kaplan K, Anglin R, et al. The gut microbiome and inflammation in obsessive-compulsive disorder patients compared to age- and sex-matched controls: a pilot study. *Acta Psychiatr Scand*. 2020;142(4):337-347. doi:10.1111/acps.13175

58. Hemmings SMJ, Malan-Müller S, van den Heuvel LL, et al. The Microbiome in Posttraumatic Stress Disorder and Trauma-Exposed Controls: An Exploratory Study. *Psychosom Med*. 2017;79(8):936-946. doi:10.1097/PSY.0000000000000512

59. Aarts E, Ederveen THA, Naaijen J, et al. Gut microbiome in ADHD and its relation to neural reward anticipation. *PloS One*. 2017;12(9):e0183509. doi:10.1371/journal.pone.0183509

60. Savin Z, Kivity S, Yonath H, Yehuda S. Smoking and the intestinal microbiome. Arch Microbiol. 2018;200(5):677-684. doi:10.1007/s00203-018-1506-2

© 2021 American Medical Association. All rights reserved.

eAppendix 7. Stool sample processing methods in the included studies

Table e7.1. Stool sample collection, storage and DNA extraction procedures of the included studies.

Study	Collection & handling by participant	Long-term storage	DNA extraction method
Naseribafrouei et al. 2014	Outpatient samples frozen at −20 °C in home freezer upon collection and transported at below zero. Inpatients stored directly at -70 °C.	at −70 °C until use	MagTM mini kit (LGC), following the manufacturer recommendations
Jiang et al. 2015	Sterile plastic cups were used to collect samples and were kept in an icebox	at −80 °C within 15 min of collection, until use	QIAamp® DNA Stool Mini Kit (QIAGEN), following manufacturer instructions, with additional glass-bead beating steps on a Mini-beadbeater (FastPrep; Thermo Electron Corp)
Aizawa et al. 2016	collected with RNA stabilizer an stored at room temperature or at 4°C until sent to lab	-	Total RNA fractions were extracted (Yakult Central Institute), method not reported
Kelly et al. 2016	Collected in plastic containers containing an anaerobic generator AnaeroGen Compact Oxoid sachet	Homogenized, aliquoted and stored at −80 °C until further use	QIAamp DNA Stool Mini Kit (QIAGEN)
Liu et al. 2016	-	Immediately stored at −80 °C until use	PowerSoil DNA Isolation Kit (MoBio), following Human Microbiome Project recommendations
Zheng et al. 2016	-	Immediately stored at −80 °C until use	PowerSoil DNA Isolation Kit (MoBio), following standard protocols
Lin et al. 2017	-	Immediately stored at −70 °C until use	Tiagen DNA Stool Mini Kit (Tiagen Biotech), following manufacturer protocols
Chen et al. 2018a	-	at −80 °C until use	PowerSoil DNA kit following standard protocol
Chen et al. 2018b	Sterile plastic cups were used to collect samples	at −80 °C until use	Tandem mass spectra were extracted and analyzed using Mascot (Matrix Science) against a combined Swiss prot-human (20151226) and TrEMBL bacteria database
Huang et al. 2018	sterile containers were used to collect samples	Immediately stored at −80 °C until use	PowerSoil DNA Kit (Missouri Biotechnology Association)
Chahwan et al. 2019	kept on ice or refrigerated before delivery to study staff	Samples placed at 4 °C and aliquots stored at −80 °C within several days	PowerFecal DNA Isolation Kit (MoBio), following manufacturer instuctions

© 2021 American Medical Association. All rights reserved.

Study	Collection & handling by participant	Long-term storage	DNA extraction method
Valles-Colomer 2019	Frozen at -18°C at home and cool transported to collection point	stored at -18°C until transport on dry ice to the research facility for -80°C storage	PowerMicrobiome RNA Isolation kit (MoBio Laboratories)
Chung et al. 2019	delivered in 4 °C to staff	and −80 °C until use	QIAamp DNA Stool Mini Kit (QIAGEN) or phenol–chloroform extraction method
Lai et al. 2019	-	immediately stored at −80 °C until use	StoolGen DNA kit (CWBiotech Co)
Rong et al. 2019	-	immediately stored at −80 °C until shipped to lab	StoolGen DNA kit (CWBiotech Co)
Chen et al. 2020	-	-	standard PowerSoil kit protocol
Chen et al. 2021	Sterile plastic cups were used to collect samples	stored at −80 °C within 30 min of collection until use	Qiagen QIAamp DNA Stool Mini Kit (Qiagen) according to manufacturer's instructions
Yang et al. 2020	-	-	E.Z.N.A. Soil DNA Kit (Omega Bio-tek, Norcross, GA, USA) according to manufacturer's instructions
Liu et al. 2020	OMNIgene•GUT stool collection kits	at −80 °C until use	ZymoBIOMICS 96 DNA Kit (Zymo Research) according to manufacturer's instructions
Stevens et al. 2020	collected with OMNIgeneGUT fecal collection kits (DNAGenotek, Ontario, Canada)	at −80 °C until use	Stool Extraction Kit following the manufacturer's instructions (Omega Bio-tek, Doraville, CA).
Stevens et al. 2018 [	-	at −80 °C until use	-
Vinberg et al. 2019	stool collection kits (Sarsted Kit sterile)	at −80 °C within 24-72 hours, until use	NucleoSpin 96 Soil kit (Macherey-Nagel)
Jiang et al. 2020	collected in a sterile plastic cup at the hospital and refrigerated	stored at −80 °C within 30 min of collection until use	FastDNATM SPIN Kit for Feces (MP Biomedicals) according to manufacturer's instructions
Evans et al. 2017	Home stool collection kits (DNA Genotek, Ontario CA)	at −80 °C until use	PowerMag soil isolation kit (MoBio)
Painold et al. 2018	-	- 20°C until use	PowerLyzer PowerSoil DNA Isolation Kit (MoBio)
Aizawa et al. 2019	collected with RNA stabilizer and stored at 4°C at home	at 4°C until use	Total RNA fractions were extracted (Yakult Central Institute), method not reported
Coello et al. 2019	collected with OMNIgeneGUT fecal collection kits (DNAGenotek, Ontario, Canada)	at −80 °C until use	NucleoSpin ® 96 Soil kit

© 2021 American Medical Association. All rights reserved.

Study	Collection & handling by participant	Long-term storage	DNA extraction method
McIntyre et al. 2019	Collected in sterile screw-capped sample jar and frozen in home freezer	Processed upon receipt; back-ups stored at −80 °C	Using in-house protocol on a MagMax™ robot (Thermo Fischer Scientific,WalthamMass)
Hu et al. 2019		at -80°C within 30 min of collection, until use	PSP Spin Stool DNA Plus Kit (Stratec, Germany) according to the manufacturer's instructions.
Lai et al. 2021	-	At -80 until use	StoolGen DNA kit (CWBiotech Co., Beijing, China)
Lu et al. 2019	-	at -80°C within 30 min of collection, until use	Qiagen Stool Kit (Qiagen, Hilden, Germany), according to a modified protocol for cell lysis
Nguyen et al. 2019	home stool collection kits (BD SWUBE Dual Swab Collection System; BD Worldwide	at −80 °C until use	Earth Microbiome Project (EMP), modified from MagAttract® PowerSoil® DNA KF Kit
Schwarz et al. 2018	Collected in a larger sampling bowl, from which duplicate samples were transferred to smaller tubes	at −80 °C until use	FastDNA Spin Kit for Soil (QBIOgene
Shen et al. 2018	-	at −80 °C until use	PowerSoil DNA kit (MoBio)
Yuan et al. 2018	-	-	QIAamp Fast DNA Stool Mini Kit (QIAGEN)
Zheng et al. 2019	-	immediately stored at −80 °C until use	QIAamp DNA Stool Mini Kit (QIAGEN, Hilden, Germany).
Pan et al. 2020	Collected and immediately transported to lab with ice packs	at −80 °C until use	E.Z.N.A. soil kit (Omega Bio-tek, Norcross, GA, U.S.) according to manufacturer's instructions
Zhu et al. 2020	-	-	-
Li et al. 2020	-	at −80 °C until use	MOBIO PowerSoil DNA Isolation Kit 12,888–100 protocol
Ma et al. 2020	collected in sterile plastic containers	immediately stored at −80 °C until use	QIAamp DNA Mini Kit (QIAGEN, Hilden, Germany) according to the manufacturer's instructions
Zhang et al. 2020	Sterile plastic cups were used to collect samples and were kept in an icebox	at −80 °C within 30 min of collection, until use	FastDNA™ SPIN Kit for Feces (MP Biomedical Inc., Santa Ana, CA, USA) according to the manufacturer's instructions with additional glass-bead beating steps
Xu et al., 2020	Collected in disposable sterile potty and transferred to tubes then frozen at -20°C	Same or next day moved to −80 °C until use	StoolGen fecal DNA extraction kit (CWBiotech, Beijing, China)
Jiang et al. 2018	Sterile plastic cups were used to collect samples and were kept in an icebox	at −80 °C within 30 min of collection, until use	QIAamp DNA Stool Mini Kit (QIAGEN), following manufacturer instructions, with the addition of a glass-

© 2021 American Medical Association. All rights reserved.

Study	Collection & handling by participant	Long-term storage	DNA extraction method
			bead beating step on a Mini-beadbeater (FastPrep; Thermo Electron Corp)
Chen et al. 2019	Sterile plastic cups were used to collect samples	at −80 °C within 15 min of collection, until use	QIAamp DNA Stool Mini Kit (QIAGEN), following manufacturer instructions
Mason et al. 2020	Frozen in home freezer after collection	at −80 °C until use	Crude DNA extracts were treated with RNAseA (QIAGEN) and column-purified (PCR Purification Kit, QIAGEN)
Armougom et al. 2009	-	-	NucleoSpinH Tissue Mini Kit according to manufacturer’s instructions
Million et al. 2013	collected using sterile plastic containers and transported “as soon as possible” to the lab	at -80°C until use	NucleoSpin® Tissue Mini Kit (information taken from cross-referenced source [1]
Kleiman et al. 2015	collected by nurses trained in collection protocols	at -80°C until use	Qiagen DNeasy® Blood and Tissue extraction kit (Qiagen, Valencia, CA, USA)
Morita et al. 2015	Collected in tubes containing the RNA stabilizer	at 4°C until use	Total RNA fractions were extracted (Yakult Central Institute), method not reported
Mack et al. 2016	Collected with a stool-collecting kit (Süsse Labortechnik, Gudensberg, Germany) from eight different sites of the stool	immediately stored at −80 °C until use	PSP Spin Stool Kit (Stratec Molecular, Berlin, Germany) according to the manufacturers’ instructions
Mörkl et al. 2017	Collected with the PSP spin stool DNA stool collection kit (Stratec, Birkenfeld, Germany)	immediately stored at −20 °C until use	PowerLyzer PowerSoil DNA Isolation Kit (MO BIO Laboratories, CA) according to the manufacturer’s instructions.
Borgo et al. 2017	-	at -80°C until use	Spin stool DNA kit (Stratec Molecular, Berlin, Germany), according to the manufacturer's instructions
Hanachi et al. 2018	-	-	Standard Operating Procedure 07 of the IHMS
Hata et al. 2019	Collected and sealed in a plastic bag containing a disposable oxygen-absorbing and carbon dioxide–generating agent and transported on ice to the laboratory within several hours.	-	-
Monteleone et al. 2021	-	at -80°C until use	PowerSoil DNA isolation kit (Qiagen, Germantown, MD, USA)
Domenech et al. *pre-print*	collected in Stool Collection Tubes (Stratec 14 Molecular)	At -20°C until use	PSP Spin Stool 15 DNA Basic Kit (Stratec Molecular)

© 2021 American Medical Association. All rights reserved.

Study	Collection & handling by participant	Long-term storage	DNA extraction method
Turna et al. 2020	collected and transferred into a sterile screw capped sample jar and placed in a household freezer (for up to 1 week)	-	-
Hemmings et al. 2017	-	-	PSP® Spin Stool DNA Plus Kit (STRATEC Molecular, Birkenfeld, Germany) according to the manufacturer's protocol 2 ("Isolation of total DNA from 1.4 ml stabilized stool homogenate with enrichment of bacterial DNA").
Aarts et al. 2017	pea-sized amount stored it in a 50ml Falcon tube, then stored at 4°C until delivery to site	At -80°C within 24hrs, until use	DNeasy1Blood and Tissue Kit (Qiagen, Venlo, The Netherlands)

– information not available

eReferences not included elsewhere:

1. Dridi B, Henry M, El Khéchine A, et al. High Prevalence of Methanobrevibacter smithii and Methanosphaera stadtmanae Detected in the Human Gut Using an Improved DNA Detection Protocol. *PLoS One*; 4. Epub ahead of print 17 September 2009. DOI: 10.1371/journal.pone.0007063.

© 2021 American Medical Association. All rights reserved.

eAppendix 8. Publication bias assessment for the alpha diversity meta-analyses

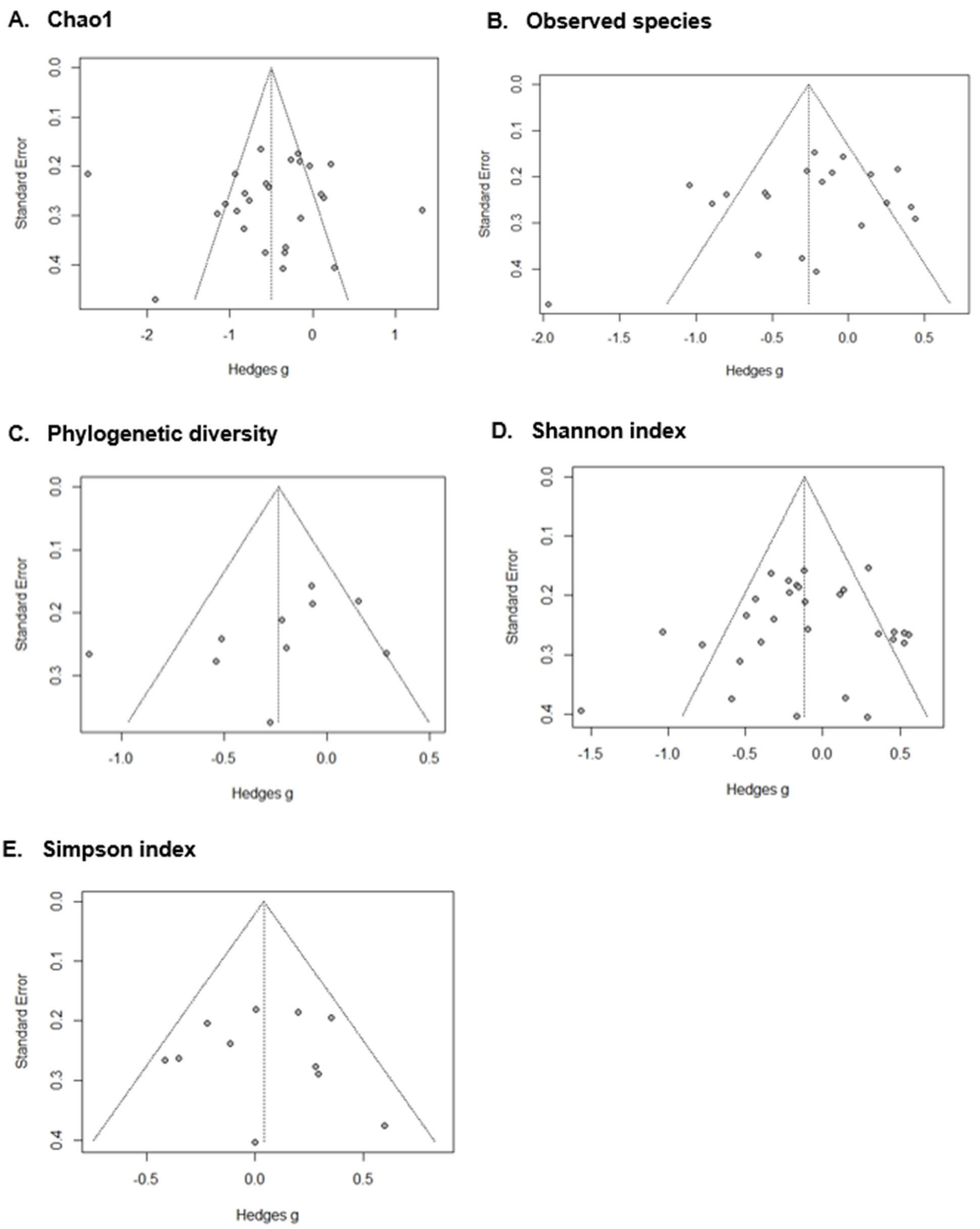

Figure e8.1. Funnel plots assessing publication bias in the meta-analyses of alpha diversity. **A**. Chao1, **B**. Observed species**, C**. Phylogenetic diversity, **D**. Shannon, **E**. Simpson index.

© 2021 American Medical Association. All rights reserved.

eAppendix 9. Beta diversity

Table e9.1. Methodology and findings of the included studies assessing beta diversity for the patient vs. control group comparison.

Disorder	Study	Year	Metric	Analysis	Finding
MDD	Jiang	2015	Unweighted Unifrac	-	no sig. difference
MDD	Kelly	2016	Bray-Curtis<br>Unweighted Unifrac<br>Weighted Unifrac	PCoA, Adonis<br>PERMANOVA	sig. different<br>sig. different<br>sig. different
MDD	Zheng	2016	Bray-Curtis	PCoA	sig. different
MDD	Lin	2017	Weighted Unifrac	PCoA	sig. different
MDD	Chen	2018a	Unifrac (nr)	PCoA, PLS-DA	sig. different
MDD	Huang	2018	Unweighted Unifrac<br>Weighted Unifrac	PCoA	sig. different<br>sig. different
MDD	Chawan	2019	Weighted Unifrac	PCoA,<br>PERMANOVA	no sig. difference
MDD	Chung	2019	Weighted Unifrac	PERMANOVA	sig. different
MDD	Lai	2019	Bray-Curtis	PCoA,<br>PERMANOVA	sig. different
MDD	Chen	2020	-	OPLS-DA	sig. different
MDD	Chen	2021	Unweighted Unifrac<br>Weighted Unifrac	PCoA	sig. different<br>sig. different
MDD	Yang	2020	Bray-Curtis	PERMANOVA	sig. different
MDD	Liu	2020	Unweighted Unifrac<br>Weighted Unifrac<br>Bray - Curtis	PCoA	sig. different<br>sig. different<br>sig. different
MDD	Stevens	2020	Bray-Curtis (unfiltered data)<br>Bray-Curtis (filtered data)	PERMANOVA	no sig. difference<br>sig. different
MDD, ANX, MDD +ANX	Mason	2020	Weighted Unifrac	PCoA	no sig. difference
MDD,<br>BD	Jiang	2020	Unweighted Unifrac<br>Weighted Unifrac<br>Bray - Curtis	PCoA	no sig. difference<br>no sig. difference<br>sig. different
MDD, BD	Rong	2019	Bray-Curtis	PCoA	no sig. difference
MDD+BD	Vinberg	2019	Generalized UniFrac	PCoA,<br>PERMANOVA	no sig. difference
BD	Evans	2017	Yue & Clayton distance	PCoA, AMOVA	sig. different
BD	Painold	2018	Unweighted Unifrac<br>Weighted Unifrac	PCoA	no sig. difference
BD	Coello	2019	Weighted Unifrac<br>Unweighted Unifrac	-	no sig. difference<br>sig. different
BD	Mcintyre	2019	Bray–Curtis	PCoA	no sig. difference
BD	Hu	2019	Unweighted Unifrac<br>Weighted Unifrac	PCoA	sig. different
BD	Lai	2021	Bray-Curtis	PERMANOVA	sig. different
SCZ	Nguyen	2019	Unweighted Unifrac<br>Bray-Curtis	PCoA	sig. different<br>sig. different
SCZ	Shen	2018	Unweighted Unifrac	PCoA, ANOSIM	sig. different
SCZ	Zheng	2019	-	PLS-DA	sig. different
SCZ	Pan	2020	Unweighted Unifrac	PCoA, ANOSIM	no sig. difference

© 2021 American Medical Association. All rights reserved.

SCZ	Zhu	2020	Bray-Curtis	-	sig. different
SCZ	Li	2020	Bray-Curtis	PCoA, PERMANOVA	sig. different
SCZ	Ma	2020	Unweighted Unifrac<br>Weighted Unifrac	PCoA, PERMANOVA	sig. different<br>no difference
SCZ	Zhang	2020	Unweighted Unifrac<br>Weighted Unifrac<br>Bray-Curtis	PCoA	sig. different<br>sig. different<br>sig. different
SCZ	Xu	2020	-	NMDS	sig. different
ANX	Jiang	2018	UniFrac unweighted	PCoA, PERMANOVA	sig. different
ANX	Chen	2019	Unweighted Unifrac<br>Weighted Unifrac	PCoA	sig. different<br>sig. different
AN	Kleiman	2015	Unweighted Unifrac<br>Weighted Unifrac	PCoA	no sig. difference<br>no sig. difference
AN	Mack	2016	Unweighted Unifrac<br>Weighted Unifrac<br>Bray Curtis	PCoA	sig. different<br>sig. different<br>sig. different
AN	Mörkl	2017	Unweighted Unifrac<br>Weighted Unifrac	PCoA, ANOSIM, Adonis	sig. different<br>sig. different
AN	Borgo	2017	-	RDA, ANOSIM	no sig. difference
AN	Hanachi	2018	Unweighted Unifrac<br>Weighted Unifrac	PCoA	sig. different<br>sig. different
AN	Montelione	2020	-	NMDS, PERMANOVA	no sig. difference
PTSD	Hemmings	2017	Unweighted Unifrac<br>Weighted Unifrac<br>Bray-Curtis	PCoA, ANOSIM	no sig. difference<br>no sig. difference<br>no sig. difference
OCD	Domenech	*pre-print*	Unweighted Unifrac<br>Weighted Unifrac<br>Bray-Curtis<br>Jensen-Shannon<br>Canberra	PCoA, PERMANOVA	no sig. difference<br>no sig. difference<br>no sig. difference<br>no sig. difference<br>no sig. difference
OCD	Turna	2020	Unweighted Unifrac<br>Weighted Unifrac<br>Bray-Curtis<br>Jaccard	PCoA, PERMANOVA	no sig. difference<br>no sig. difference<br>no sig. difference<br>no sig. difference

PCoA = principal coordinates analysis; PERMANOVA = permutational analysis of variance; PLS-DA = principal least squares discriminant analysis; OPLS-DA = orthogonal principal least squares discriminant analysis; NMDS = non-metric multidimensional scaling

© 2021 American Medical Association. All rights reserved.

eAppendix 10. Figures for study-level findings of relative abundance of microbial taxa

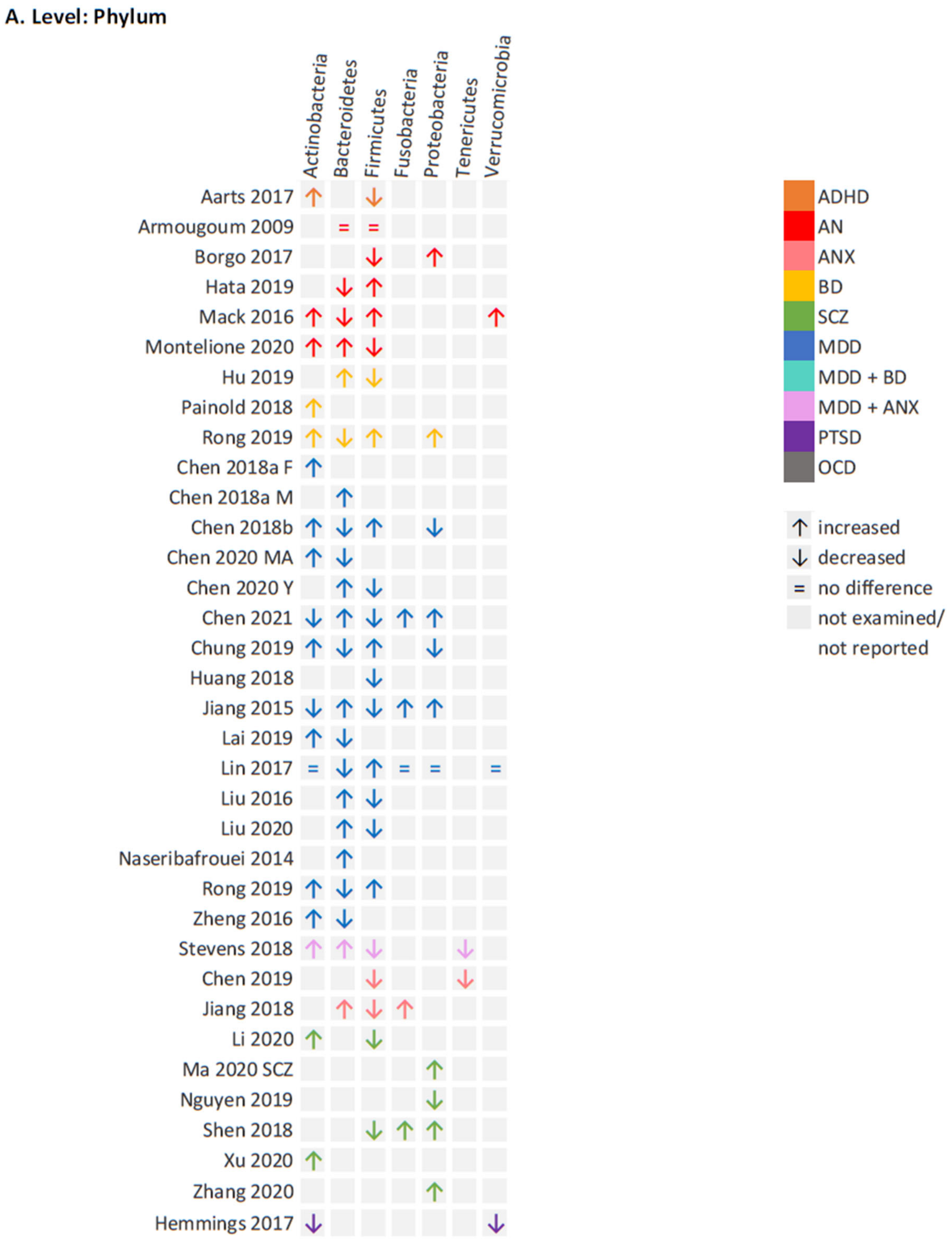

Figure e10.1. Study-level findings of relative abundance of microbial taxa in patients with psychiatric disorders compared to healthy controls at the: A. Phylum level, B. Family level, C1-2. Genus level. ADHD= attention deficit hyperactivity disorder, AN= anorexia nevrosa, ANX= anxiety, BD= bipolar disorder, MDD= depression, OCD= obsessive compulsive disorder, PTSD= post-traumatic stress disorder, SCZ= psychosis & schizophrenia

© 2021 American Medical Association. All rights reserved.

B. Level: Family

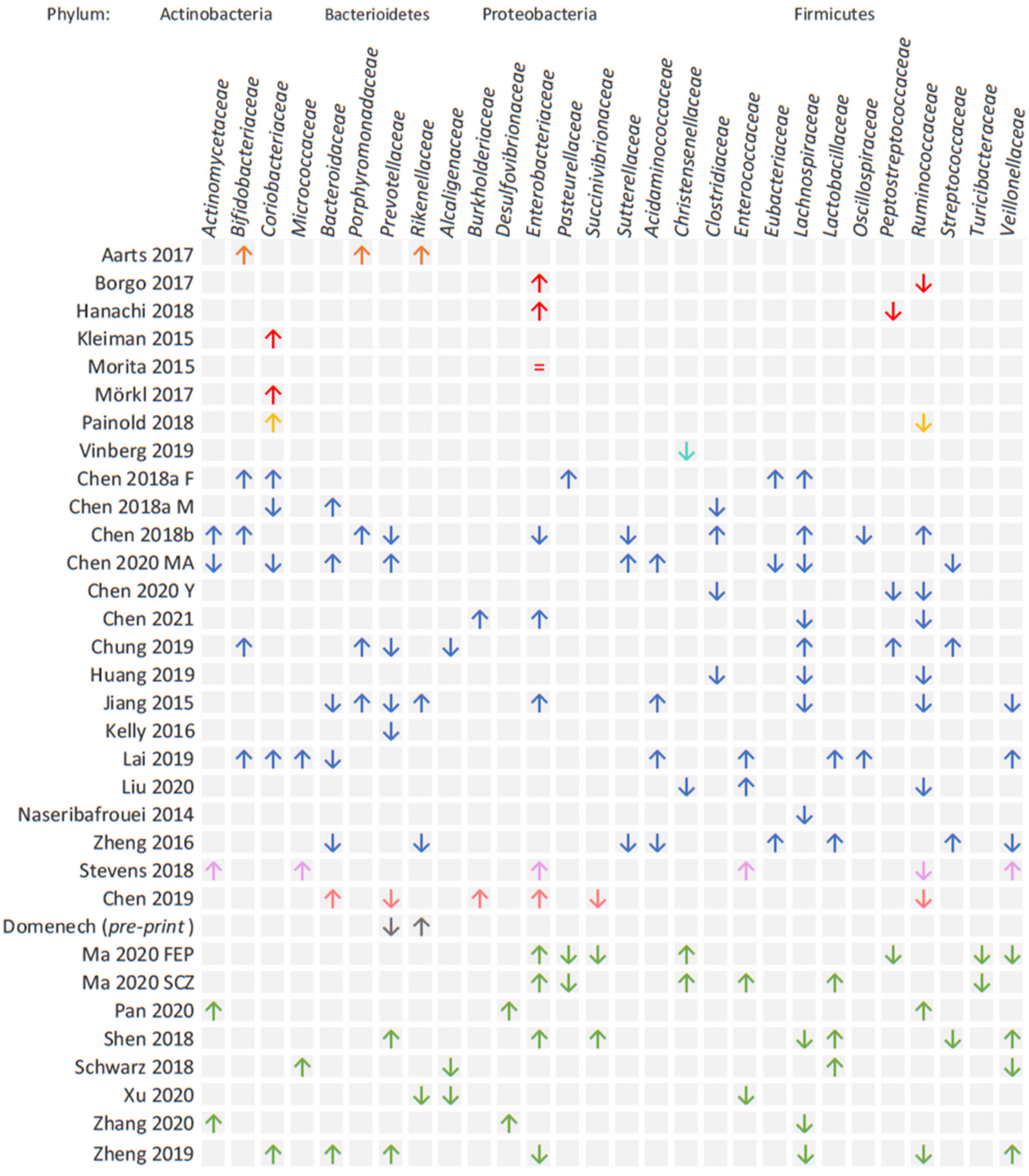

Figure e10.1. *Continued*

© 2021 American Medical Association. All rights reserved.

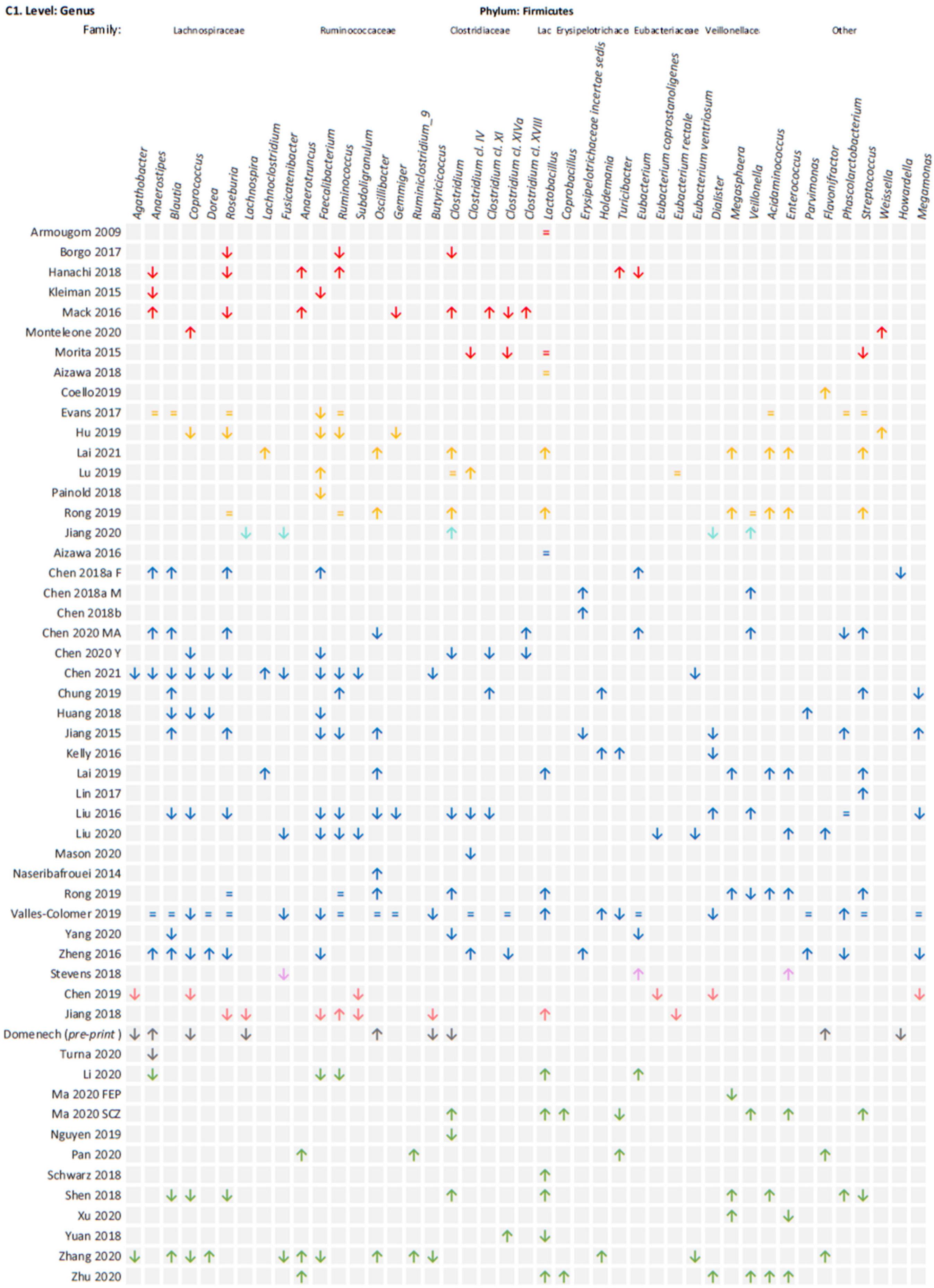

Figure e10.1. *Continued*

© 2021 American Medical Association. All rights reserved.

C2. Level: Genus

Phylum:	Actinobacteria							Bacteroidetes						Proteobacteria										Other		
	Actinomyces	*Bifidobacterium*	*Atopobium*	*Collinsella*	*Eggerthella*	*Olsenella*	*Slackia*	*Alistipes*	*Bacteroides*	*Odoribacter*	*Parabacteroides*	*Paraprevotella*	*Prevotella*	*Bilophila*	*Desulfovibrio*	*Escherichia-Shigella*	*Citrobacter*	*Enterobacter*	*Klebsiella*	*Salmonella*	*Parasutterella*	*Sutterella*	*Haemophilus*	*Succinivibrio*	*Fusobacterium*	*Methanobrevibacter*
Aarts 2017		↑			↑					↑	↑															
Armougom 2009																										↑
Borgo 2017																										↑
Hanachi 2018																			↑	↑						
Mack 2016		↑																								↑
Million 2013																										↑
Monteleone 2020											↓															
Morita 2015		=	=										=													
Aizawa 2018		=																								
Evans 2017		=		=				=	=		=		=								=					
Hu 2019									↑		↑										↓					
Lai 2021		↑	↑		↑	↑	↑		↓						↑	↑			↑							
Lu 2019		=	↑															↑								
Rong 2019		↑			↑			=	↑	↓	=		=		↑	↑		↑	↑	↑			↓			
Jiang 2020																						↓				
Aizawa 2016		↓																								
Chen 2018a F	↑	↑	↑		↑	↑									↑							↓				
Chen 2018a M			↑						↑																	
Chen 2020 MA	↑				↑																	↓				
Chen 2020 Y									↓																	
Chen 2021		↓									↑					↑										
Chung 2019		↑			↑						↑		↓									↓				
Jiang 2015								↑	↓		↑		↓								↑					
Kelly 2016					↑							↑	↓													
Lai 2019		↑	↑		↑	↑	↑		↓																	
Lin 2017													↑						↑							
Liu 2016		↓		=				↓	↑	↓	↓	↑	↑			↓					=	↓	↑		↓	
Liu 2020															↓											
Naseribafrouei 2014								↑																		
Rong 2019		↑			↑			=	↑	↓	=		=		↑	=		=	=	=			↓			
Valles-Colomer 2019	=	=		=	=	=		=	=		↑	=	=			=					=	=				
Yang 2020									↑																	
Zheng 2016				↑		↑		↓																		
Stevens 2019	↑			↑			↑																			
Chen 2019									↑				↓			↑										
Jiang 2018									↑							↑						↓			↑	
Domenech (*pre-print*)								↑																		
Turna 2020										↓																
Li 2020				↑																				↑		
Ma 2020 FEP																↑									↓	
Ma 2020 SCZ									↓		↓					↑		↑								
Nguyen 2019																						↓	↓			
Pan 2020	↑													↑												
Shen 2018				↑									↑		↑		↑		↑					↑		↑
Xu 2020					↑																					
Yuan 2018		↓							=							↓										
Zhang 2020	↑				↑								↑	↑							↑					
Zhu 2020		↑															↑									↑

Figure e10.1. *Continued*

© 2021 American Medical Association. All rights reserved.
